# Rational design of tunable pH switches through shadow-strand hybridization-actuated displacement engineering

**DOI:** 10.1093/nar/gkaf849

**Published:** 2025-09-05

**Authors:** Xiaole Han, Xiangyu Dong, Xiaomei Lin, Hongyan Yu, Li Zhang, Weitao Wang, Yaoyi Zhang, Jianbo Jiang, Xingyu Liu, Gang Yang, Yongcan Guo, Guoming Xie

**Affiliations:** Key Laboratory of Clinical Laboratory Diagnostics (Chinese Ministry of Education), College of Laboratory Medicine, Chongqing Medical University, Chongqing 400016, P. R. China; Key Laboratory of Clinical Laboratory Diagnostics (Chinese Ministry of Education), College of Laboratory Medicine, Chongqing Medical University, Chongqing 400016, P. R. China; Key Laboratory of Clinical Laboratory Diagnostics (Chinese Ministry of Education), College of Laboratory Medicine, Chongqing Medical University, Chongqing 400016, P. R. China; Key Laboratory of Clinical Laboratory Diagnostics (Chinese Ministry of Education), College of Laboratory Medicine, Chongqing Medical University, Chongqing 400016, P. R. China; Key Laboratory of Clinical Laboratory Diagnostics (Chinese Ministry of Education), College of Laboratory Medicine, Chongqing Medical University, Chongqing 400016, P. R. China; Key Laboratory of Clinical Laboratory Diagnostics (Chinese Ministry of Education), College of Laboratory Medicine, Chongqing Medical University, Chongqing 400016, P. R. China; Key Laboratory of Clinical Laboratory Diagnostics (Chinese Ministry of Education), College of Laboratory Medicine, Chongqing Medical University, Chongqing 400016, P. R. China; Key Laboratory of Clinical Laboratory Diagnostics (Chinese Ministry of Education), College of Laboratory Medicine, Chongqing Medical University, Chongqing 400016, P. R. China; Key Laboratory of Clinical Laboratory Diagnostics (Chinese Ministry of Education), College of Laboratory Medicine, Chongqing Medical University, Chongqing 400016, P. R. China; Department of Neurosurgery, The First Affiliated Hospital of Chongqing Medical University, Chongqing 400016, P. R. China; Clinical Laboratory of Traditional Chinese Medicine Hospital Affiliated to Southwest Medical University, LuZhou Key Laboratory of Nanobiosensing and Microfluidic Point-of-Care Testing, Luzhou 646000, P. R. China; Key Laboratory of Clinical Laboratory Diagnostics (Chinese Ministry of Education), College of Laboratory Medicine, Chongqing Medical University, Chongqing 400016, P. R. China

## Abstract

Local pH variations play a pivotal role in numerous critical biological processes. However, achieving the tunability and selectivity of pH detection remains a challenge. Here, we present a DNA-based strategy that enables programmable and selective pH responses, which is termed shadow-strand hybridization-actuated displacement engineering (SHADE). The tunability of pH responses is accomplished via rational manipulations of shadow strands derived from the i-motif-forming sequence. Shadow strands, which can be considered the “nucleic acid molecular chaperones,” assist in the folding of i-motif structure under acidic conditions via toehold-mediated strand displacement reactions (TMSDR). The response to alkaline conditions is achieved through a hairpin shadow (HS) containing A^+^-C pairs in stem. Combining i-motif-forming sequence and HS allows for the development of a narrow pH-responsive probe. Furthermore, aptamer was conjugated to guide the probe on cell surfaces. The pH sensitivity of SHADE allows for significant fluorescence enhancement in acidic tumor microenvironments, thereby improving the signal-to-noise ratio *in vivo* imaging. This work represents the application of TMSDR programmability to pH regulation. The SHADE strategy holds promise beyond pH sensing, potentially enabling the manipulation of diverse quadruplex architectures and facilitating the creation of highly responsive components for synthetic molecular devices and signal transduction networks.

## Graphical Abstract

**Graphical Abstract:**
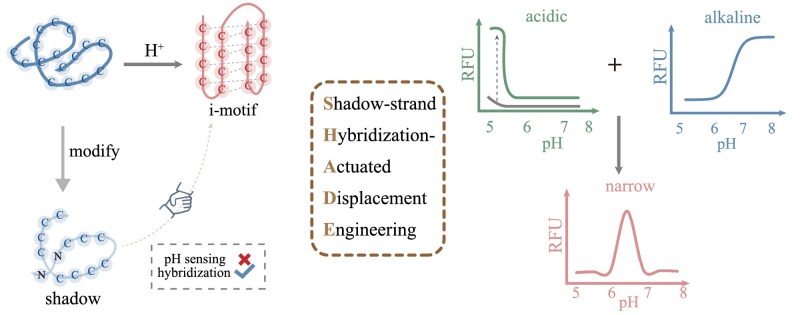

## Introduction

Physiological pH conditions are meticulously maintained through stringent homeostatic control within cells and tissues. Local pH variations play a critical role in numerous essential biological processes [1, 2], ranging from enzymatic catalysis [3, 4] and protein folding [5] to membrane function [6, 7] and cellular apoptosis [8]. Due to these reasons, there is significant interest in engineering molecular systems that exhibit programmable behavior in response to subtle pH changes [9]. Such systems hold great potential for a variety of applications, including *in vivo* imaging, clinical diagnostics, and drug delivery [10–12], enabling precise control of molecular devices for a wide range of clinical uses [13]. DNA is a natural material. Owing to ease of synthesis, high programmability, intelligent responsiveness, and excellent biocompatibility [14, 15], DNA has been extensively utilized in molecular assembly to create a variety of complex nanostructures [16], which have been applied in biodiagnosis [17–19], biotherapeutics [20], nanorobotics [21, 22], biocomputing [23], and beyond. DNA nanostructures can be triggered to transition between defined states through chemical or physical stimulation [12, 24]. I-motif structure, as a secondary DNA structure, exhibits remarkable advantages in pH sensing applications. One of its most distinctive features is its sensitivity to acidic environments, where the structural stability is significantly enhanced at lower pH conditions [9, 12]. Several research groups have developed pH-triggered i-motif structure-based probes or nanodevices [25–32]. These probes always design blockers that are complementary to i-motif-forming sequences to promote the formation of duplexes under alkaline conditions. Under acidic conditions, i-motif-forming sequence folds and dissociates with blocker to trigger downstream reactions or promote self-assembly of nanomachines [32–35]. Other probes split the i-motif-forming sequence into two single-stranded nucleic acids. Under acidic conditions, i-motif structure is formed through the interaction between the two strands to generate high fluorescence resonance energy transfer signal or mediate self-assembly of nanoparticles [24, 30]. While some of these structures exhibit promising and advantageous features, such as rapid response and sustained efficiency over multiple cycles, a notable limitation inevitably affects their performance—they operate only within a fixed pH window [36]. Adjusting pH sensitivity is always achieved through changing the length and complementary strength of the blocker. This regulation is limited. More importantly, these strategies mostly lack pH selectivity, with “all or nothing” pH response characteristic.

Molecular chaperones represent a class of proteins that facilitate the proper folding, assembly, transport, and degradation of other proteins [37–39]. Drawing inspiration from this biological mechanism, shadow strand of i-motif-forming sequence was designed. These synthetic constructs share sequence similarity with the i-motif-forming sequence but lack consecutive cytosine (C) bases, thereby maintaining hybridization affinity with blocker at low pH response. Shadow strands function to assist i-motif-forming sequence in dissociating from the duplex, promoting the formation of characteristic secondary structure under acidic conditions. Toehold-mediated strand displacement reaction (TMSDR) is a fundamental process in DNA nanotechnology, wherein an invading strand binds to a complementary single-stranded domain (termed the toehold) on a DNA substrate, subsequently displacing one or more pre-hybridized strands through branch migration (BM) [40, 41]. This mechanism has emerged as a powerful and programmable tool for constructing complex molecular systems with dynamic behaviors in the field of dynamic DNA nanotechnology. While previous studies have demonstrated pH-dependent activation or inhibition of strand displacement [42, 43], a comprehensive strategy for controlling pH sensitivity through TMSDR has remained elusive. Herein, we present a rational design and programming approach for precisely tuning pH-responsive ranges by shadow-strand Hybridization-actuated displacement engineering (SHADE). Shadow strands is a new regulatory factor. pH response profiles can be regulated through different shadow strands, which affect the TMSDR between the shadow strand and the duplex formed by i-motif-forming sequence and blocker. Based on single-strand shadow, complementary domains with pH-sensitive A^+^-C pair [44] were introduced to design hairpin shadow (HS) for alkaline response. Under acidic conditions, a base is protonated. The stability of a duplex DNA containing an A-C increased by ∼1.4 kcal/mol, in accord with a gain of a single hydrogen bond [45]. The stable A^+^-C pair locks the toehold domain of HS, preventing TMSDR.

While extracellular acidosis has emerged as a diagnostic hallmark of tumors in clinical practice, imaging the acidic tumor microenvironment remains challenging. The minimal pH difference between tumor extracellular environment and normal tissues, along with the acidic lysosomal environment in normal cells, complicates specific detection. The pH response strategy based on i-motif structure is usually difficult to selectively target the slightly acidic tumor microenvironment. The one-size-fits-all acidic response capability needs to be improved. Since the acid-sensitive and alkali-sensitive strategies described earlier are non-interfering, we developed a narrow-range pH-responsive probe, showing optimal fluorescence at pH 6.5. When pH is too high or low, the fluorescence signal is weak. Furthermore, the modular nature of DNA allows aptamers to be incorporated for targeting cell surface without interfering with the pH response function. We validated this SHADE strategy in HeLa cells, demonstrating its capability for extracellular acidosis detection. The system also enables noninvasive imaging of solid tumors *in vivo*.

## Materials and methods

### Materials

DNA oligonucleotides used in this study were purchased from Sangon Biotech Co. (Shanghai, China). All DNA oligonucleotides were purified via high-performance liquid chromatography. All sequences are presented in Supplementary Tables S1–S5. I-motif-forming sequence was modified with Cy5. Different blocker strands were modified with BHQ2. HEPES, MgCl_2_, NaAc, Tris–HCl, and NaCl were purchased from Sangon Biotech Co. (Shanghai, China). WST-1 was purchased from Takara Bio USA (San Jose, CA, USA). HeLa cells were purchased from Shanghai Institutes for Biological Sciences, Chinese Academy of Science.

### Instrumentations

Fluorescence spectra were collected using a Rotor-Gene 6000 instrument (Corbett Research, Mortlake, Australia). CD experiments were performed using a J-1500 Circular Dichroism Spectrometer (JASCO Corporation, Japan). The UV–vis absorption spectra of each sample were acquired using a UV-2550 spectrophotometer (SHIMADZU, China). Confocal microscopic images were obtained by a laser confocal microscope (Leica TCS SP8). The flow cytometry analysis was carried out on a CytoFLEX instrument (Beckman Coulter, USA). *In vivo* and *ex vivo* imaging was performed using the IVIS Lumina Series III In Vivo Imaging System (PerkinElmer, Waltham, MA).

### Buffer conditions

All DNA oligonucleotides were redissolved in ddH_2_O. The buffer used in the annealing step and TMSDR was Phy buffer, consisting of Tris–HCl (10 mM), NaAc (10 mM), HEPES (10 mM), NaCl (100 mM), and MgCl_2_ (1.25 mM). The pH of Phy buffer is adjusted by adding HCl (1M) and NaOH (1M).

### Assembly procedure

All DNA duplexes consisting of i-motif-forming sequences and blockers were assembled in Phy buffer. Duplexes or hairpins were diluted to 5 μM in Phy buffer with pH 7 and annealed in a polymerase chain reaction thermal cycler. The temperature was set at 95°C for 5 min and cooled down to 20°C by 0.1°C/s. The reactants were added to Phy buffers with different pH values for reaction.

### Time-based fluorescence acquisition

Double-stranded complexes and shadows are added in test tubes. The volume of all reactions is 20 μl. Temperature was set to 37°C except to measure the effect of temperature, and gain was set to default. Unless otherwise stated, the concentration of i-motif-forming sequence is 100 nM, and the concentrations of blocker or shadow are 150 nM. All fluorescent signals were monitored under the red channel (646 nm/664 nm). In order to determine pH^T^, the signal versus pH data points were plotted and fitted with Boltzmann sigmoidal fits using Origin 8.5.1 software. The values for “x_0_” (point of inflection) were reported as pH^T^’s. Unless otherwise specified, toehold stands for forward toehold.

### CD measurement

The duplex and HS were first incubated in the Phy buffers with different pH values for 30 min at 37°C. For each single experiment, 300 μL of 5 μM sample was used in a 0.3 cm optical path cuvette. Each curve represents the correction for the corresponding buffer blank. At pH 6.5, the i-motif structure showed a positive peak at 288 nm and a negative peak at 263 nm, which is the characteristic spectrum of i-motif structure.

### UV measurement

The duplex and HS were first incubated in the Phy buffers with different pH values for 30 min at 37°C. Then, the UV–vis absorption spectra of each sample were acquired in the range of 220–320 nm. The final concentration of all samples was 1 μM.

### Native polyacrylamide gel electrophoresis

Twenty-five milliliters of 12% polyacrylamide gels were prepared before. Five microliters sample solution was mixed with 1 μL of 6× loading buffers and then loaded onto the 12% polyacrylamide gels. The electrophoresis was conducted at 120 V for 1 h at room temperature. Photographs were performed under UV light by Bio-Rad ChemiDoc XRS imaging system (Bio-Rad Laboratories, American).

### Cell culture

HeLa cells were cultured in Dulbecco’s modified Eagle’s medium (DMEM) supplemented with 10% fetal bovine serum (WISENT, Cat: 086-150, Australian) containing 100 units of penicillin and 100 μg/mL of streptomycin in 5% CO_2_ incubators at 37°C as described.

### Tumor microenvironment recognition assay

Tumor microenvironment recognition was evaluated by flow cytometry and confocal laser scanning microscopy (CLSM) imaging. For flow cytometry, HeLa cells (5 × 10^5^) were washed in Phy buffers of different pH and incubated with 200 nM SHADE probes with different designs at room temperature for 30 min. The excess probe is then removed by washing 3 times with the corresponding pH of Phy buffer. The cells were analyzed by flow cytometry after centrifugation and suspension. For CLSM imaging, HeLa cells were seeded in a 15 mm glass-bottomed dish (1 × 10^5^ cells/dish) and incubated for 24 h. The DMEM medium was replaced with 200 μL Phy buffer of different pH containing the 200 nM SHADE probe. After 30 min of incubation, cells were washed three times and replenished with Phy at the corresponding pH for CLSM imaging.

### WST-1 cell proliferation assay

Exponentially growing cells were seeded in 96-well plates at a density of 3000 cells per well. At the indicated timepoints, the Premixed WST-1 Reagent (Takara Bio USA, San Jose, CA) was added, followed by an incubation at 37°C for 2 h, and the absorbance at 450 nm was measured using a microplate reader (Biotek, EON, USA) as described.

### Mice and tumor models

All the experiments described herein were in accordance with the regulations of the Institutional Animal Care and Use Committee (IACUC). BALB/c nude mice (male, 16–20 g) were maintained in a specific-pathogen-free animal facility with access to water and food. Primary tumor models were established by inoculating HeLa cells (2 × 10^6^ cells/100 μL in PBS) into the right shoulder of BALB/c nude mice.

### Imaging of primary tumors

Tumor length and width were measured with calipers, and the tumor volume (V) was calculated using the following equation: V = length × width × width/2. When the primary tumors grew to 200–300 mm^3^, the imaging was done following i.v. injection of the SHADE probes (Cy5 filter was used). Mice were randomly divided into three groups for the treatment of Apt-SHADE, Apt-MSHADE, or MApt-SHADE. Each probe (2 μM, 200 μL) was injected into the tumor-bearing mice through tail vein. Fluorescence is measured at the indicated time intervals. Two hours post-injection, mice were euthanized, and tumors and major organs were collected for *ex vivo* imaging. Fluorescence intensity was normalized by representative region of interest analysis. The acquired data were quantitatively analyzed using the Living Image Software (PerkinElmer) as described.

## Results and discussion

### Design of shadow-strand hybridization-actuated displacement engineering

**Figure 1.**
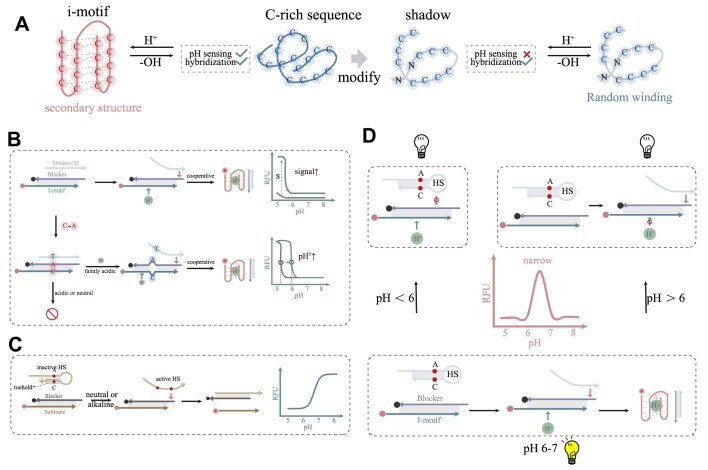
Schematic design of SHADE. (**A**) Shadow strands derived from i-motif-forming sequence. (**B**) Mechanism of SHADE. The small red arrow represents the toehold binding of TMSDR. The small green arrow indicates the interaction between hydrogen ions and i-motif-forming sequence. When A^+^-C pairs were introduced, TMSDR only occurred under faintly acidic conditions. (**C**) Design of HS strands and schematic of TMSDR activation under alkaline pH conditions. (**D**) Signal generation in mildly acidic pH conditions through the integration of i-motif and HS. Complex pH dependence is achieved through a three-state system, where i-motif formation is inhibited by stable HS at low pH or strong duplex confinement at high pH, with a small window between these extremes where i-motif is folded.

The i-motif structure formed at acidic pH consists of two parallel-stranded C:C^+^ hemiprotonated base-paired duplexes that are intercalated in an antiparallel manner. Through removal or substitution of C bases, we engineered shadow strand (Fig. 1A). Shadow strand retains the hybridization capability while losing pH-sensing properties. Conventional i-motif structure-based pH sensor typically employs complementary blocker strands. Under acidic conditions, hydrogen ions compete with the blocker for i-motif-forming sequence binding sites. When protonation forces are sufficient, the i-motif-forming sequence folds and generates a fluorescent signal. I-motif-forming sequence cannot dissociate from duplex even under acidic conditions when blocker is too long. When the i-motif-forming sequence is too short, the hybridization of duplex is unstable (Supplementary Fig. S1). Shadow strand enhances this process through its ability to hybridize with the blocker, facilitating the release of the i-motif-forming sequence and subsequent i-motif structure formation (Fig. 1B). Through TMSDR, shadow and i-motif-forming sequences establish a cooperative relationship, producing higher signals at acidic pH values. This system offers enhanced programmability for acidic pH response through the dual regulation of both blocker and shadow strands.

The A base is introduced into blocker strand, and forms the A^+^-C pair between the i-motif-forming sequence, enabling precise tuning of the folding/unfolding midpoint (pH^T^) [9]. In acidic conditions, the A^+^-C pair is stable, while hydrogen ion force is strong, and the i-motif structure is formed with the assistance of the shadow strand. Under mildly acidic conditions, the A^+^-C pair tends to be unstable. Although the hydrogen ion is weakened, the completely complementary base between shadow and blocker causes TMSDR. Under neutral or alkaline conditions, the lack of hydrogen ions makes TMSDR difficult to occur. Therefore, under mildly acidic conditions, the synergistic effect of i-motif-forming sequence and shadow is improved. This modification shifts pH^T^ toward more physiological pH values (Fig. 1B).

In the acidic response system, the i-motif-forming sequence is used to sense pH. We engineered hairpin shadow strands (HS) by introducing intramolecular complementary regions and A^+^-C pairs. Under neutral or alkaline conditions, A^+^-C dissociation exposes the toehold domain, starting TMSDR and generating fluorescence. Conversely, acidic pH inactivates the shadow strand, inhibiting TMSD (Fig. 1C). These two modifications contribute opposite pH dependencies; three-state pH dependence can be introduced (Fig. 1D). At high pH, HS forms the single strand. However, the effect of hydrogen ions on the i-motif-forming sequence is insufficient. TMSDR is not prone to occur, which leads to low fluorescence signal. Within the narrow range, the combined effect of hydrogen ions and weak A^+^-C hybridization decreases binding competition, enabling TMSDR and high fluorescence signals. Finally, as the pH is further decreased to the point where A–C mismatch becomes protonated, hairpin structure of shadow is stable, inhibiting TMSDR and leading to a low fluorescence signal.

**Figure 2.**
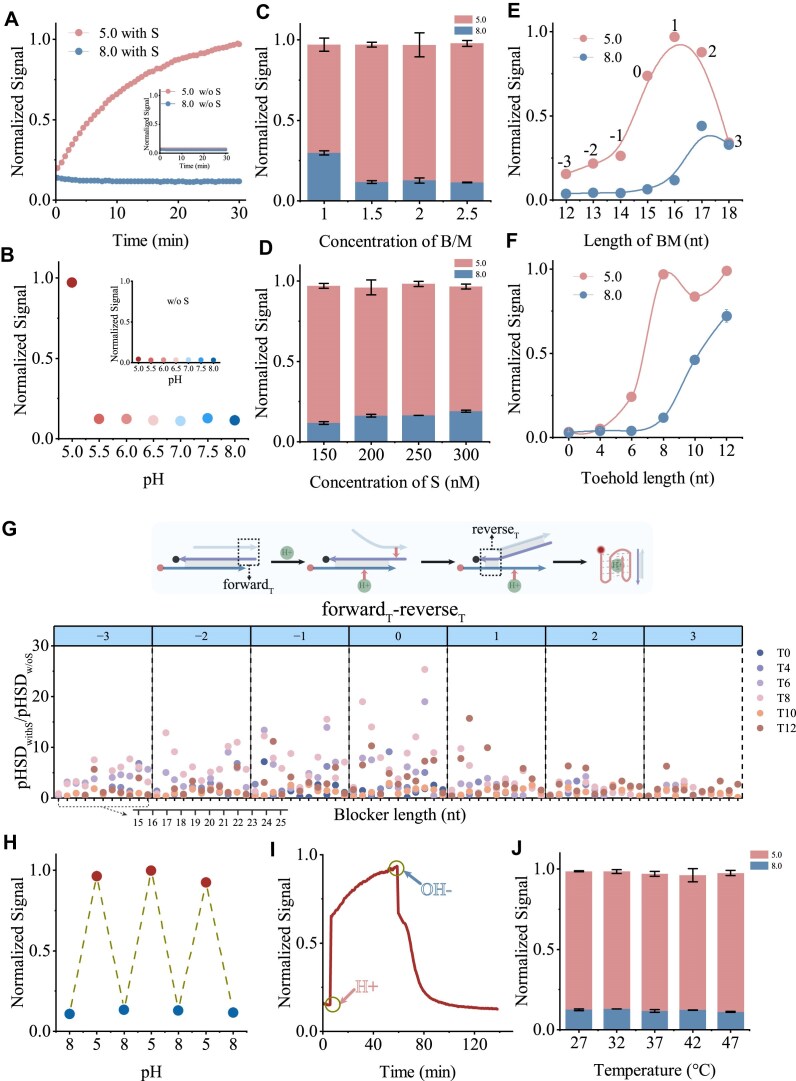
SHADE under acidic conditions. (**A**) Signal differentiation between pH 5.0 and pH 8.0 using SHADE. Inset shows signals in the absence of shadow strands. (**B**) SHADE across the pH gradient. Inset shows signals without shadow strands. (**C**) Signals at pH 5.0 and pH 8.0 with varying blocker to i-motif ratios (B/M). i-motif concentration fixed at 100 nM. The concentrations of blocker and shadow are the same. (**D**) Signals at pH 5.0 and pH 8.0 with different shadow strand concentrations. i-motif concentration fixed at 100 nM and blocker concentration fixed at 150 nM. (**E**) pH 5.0 and pH 8.0 signals with varying branch migration domain lengths. Toehold length fixed at 8 nt. The numbers on the curve represent the forward length minus the reverse toehold length (ΔfT-rT). (**F**) pH 5.0 and pH 8.0 signals with varying toehold lengths. Branch migration domain length fixed at 16 nt. (**G**) Enhancement of pH signal difference (pHSD ratio) values with varying shadow and blocker lengths. Data are categorized based on (ΔfT-rT) (blue boxes). pHSD = (F5.0 − F0)/(F8.0 − F0), where F0 represents initial fluorescence. Probe resetability was validated by endpoint fluorescence (**H**) and real-time fluorescence (**I**). (**J**) pH 5.0 and pH 8.0 signals at different temperatures. In panels (A–D), blocker is B23-t8, and shadow strand is S16-t8.

We initially investigated the role of shadow strands in pH-responsive behavior. The results revealed significantly higher fluorescence signals at pH 5.0 compared to pH 8.0 by introducing shadow strand. In contrast, negligible signals were observed at both pH values in the absence of shadow strands (Fig. 2A and B). Control experiments excluded pH effects on strand displacement reactions (Supplementary Fig. S2), demonstrating the auxiliary function of shadow in promoting i-motif structure formation. Blocker-to-i-motif-forming sequence ratios influenced fluorescence quenching efficiency. The concentration of shadow is the same as that of the blocker. Low blocker concentration will lead to increased signals at pH 8.0, and the optimal blocking is achieved at a ratio of 1:1.5 (Fig. 2C and Supplementary Fig. S3). Optimal shadow concentration was observed at equimolar concentrations with blocker strands (Fig. 2D and Supplementary Fig. S4). More shadow will promote the TMSDR at 8.0 pH, thereby increasing the fluorescence signal. Toehold and branch migration domains present critical design parameters. With a fixed 8-nt forward toehold, increasing length of branch migration domain enhanced both pH 5.0 and pH 8.0 signals due to widening toehold length differences between forward and reverse toeholds (Δf_T_-r_T_). When Δf_T_-r_T_ is 0 or 1 nt, the discrimination between 5.0 and 8.0 is better (Fig. 2E and Supplementary Fig. S5). However, 18-nt shadow (refer to 10-nt BM) showed reduced pH 5.0 signal, likely due to increased fold formation under acidic conditions. Similarly, variable toehold lengths demonstrated that short toeholds reduced pH 5.0 signal, while excessive toehold length diminished discrimination between 5.0 and 8.0 (Fig. 2F and Supplementary Fig. S6). Eight nucleotides is the better choice. These findings highlight the importance of domain design for optimal pH-responsive behavior.

To investigate the impact of TMSD on pH response, we conducted orthogonal experiments with varying lengths of shadow and blocker. Compared with no-shadow systems (Supplementary Fig. S7), signal enhancement always occurred under acidic conditions (Supplementary Figs S8–S31). This effect was quantified by calculating the ratio of pH signal difference between systems with and without shadow (pHSDwithS/pHSDw/oS) (Fig. 2G). Classification revealed optimal performance when Δf_T_-r_T_ was zero. The strongest pHSD enhancement occurred with the 8-nt forward toehold, consistent with established strand displacement principles. When toehold is 0 or 4 nt, the signals at different pH values are all weak (Supplementary Figs S8–S12). When toehold is longer than 6 nt, the signal of pH 5.0 increases significantly, and it becomes more obvious with the growth of shadow. However, when toehold is 10 or 12 nt, the signal of 8.0 increases, reducing pHSD (Supplementary Figs S22–S31). Control experiments with i-motif-forming sequence variants performed the loss of pH sensitivity, regardless of the Δf_T_-r_T_ value (Supplementary Fig. S32). Next, the resettability of the probe was demonstrated by adding acids and bases to the solution to change the pH. End-point fluorescence and real-time fluorescence were measured (Fig. 2H and I). When duplex and shadow were incubated together at pH 8.0, TMSDR was difficult to occur due to the lack of hydrogen ions, which appeared as a low fluorescence signal. Then we adjusted the DNA solution to pH 5.0 and analyzed it. The folding of the i-motif-forming sequence and the attraction between shadow and blocker jointly lead to the increase of fluorescence signal. When we readjusted the DNA solution to pH 8.0, the fluorescence decreased again. Shadow strand executed similar auxiliary folding functions at different temperatures (Fig. 2J and Supplementary Fig. S33), demonstrating the temperature robustness of SHADE.

### Design of A^+^-C pair and HS

**Figure 3.**
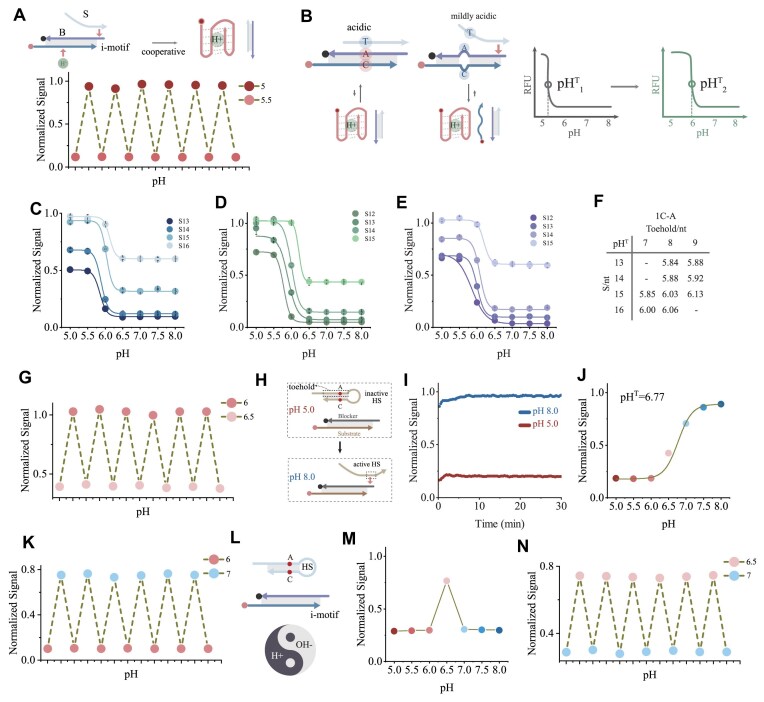
Design of A^+^-C pairs and HS strands. (**A**) Resetability of normal shade between pH 5.0 and pH 5.5. (**B**) Schematic of A^+^-C pair design between i-motif and blocker, and its impact on pH^T^. (**C**) pH response of different shadow branch migration lengths with a single A^+^-C pair. Toehold is 8 nt. pH response of different shadow branch migration lengths with two (**D**) and three (**E**) A^+^-C pairs. Toehold is 8 nt. (**F**) Variation of pHT with different branch migration and toehold lengths, derived from data in panel (C) and S35. (**G**) Resetability of 23-nt S with two A^+^-C pairs and 8-nt toehold. (**H**) Design of HS strands and (**I**) fluorescence kinetics at pH 5.0 and pH 8.0. (**J**) Signal intensity and pHT values of HS across a pH gradient. (**K**) Resetability of alkaline-preferring probes between pH 6.0 and pH 7.0. (**L**) Integration diagram of i-motif and HS. (**M**) Signal intensity of HS with 15-nt branch migration and 8-nt toehold across a pH gradient. (**N**) Resetability of the mildly acidic pH-sensitive design between pH 6.5 and pH 7.0.

While shadow strand enhanced signal at pH 5.0, discrimination of weaker acidity remains challenging. Resetability is limited to the 5.0–5.5 range (Fig. 3A), likely due to the stronger hybridization force of base pairs compared to the proton-dependent folding of i-motif-forming sequence. At pH 5.0, folding of i-motif-forming sequence requires shadow strand assistance. Above pH 5.5, weakened folding forces allow shadow-blocker hybridization to dominate, reducing pH discrimination. This operational range (5.0–5.5) limits practical applications. To shift pH^T^ toward physiological conditions, we introduced A^+^-C pairs between i-motif-forming sequence and blocker. The mildly acidic conditions weakened the stability of the A^+^-C pairs. The fully complementary base between blocker and shadow triggered the TMSDR, generating a fluorescence signal (Fig. 3B). Single A^+^-C pairs produced varying pH^T^ values depending on branch migration lengths and toehold lengths (Fig. 3C and Supplementary Figs S34 and S35). Increasing the value of Δf_T_-r_T_ progressively raised pH^T^, though excessive differences weakened discrimination between 5.0 and 8.0 (Fig. 3F). The optimal toehold length also remained 8 nt. Similar patterns emerged with two (Fig. 3D and Supplementary Figs S36–S38) and three A^+^-C pairs (Fig. 3E and Supplementary Fig. S39). The increase in the number of A^+^-C pairs has increased the pH^T^ values, with a maximum of 6.21. Furthermore, the A^+^-C pairs near the 5′ end of blocker have a better increasing effect on pH^T^ than those near the 3′ end (Supplementary Fig. S40). After comprehensively considering signal intensity and acid-base discrimination effect, these designs enabled probe resetability across pH ranges of 6–6.5, 5.5–6.5, or 5.5–6.0, demonstrating tuning pH response through DNA engineering (Fig. 3G and Supplementary Fig. S41).

In the acidic response system, the i-motif-forming sequence is used to sense pH. HS strand was designed by introducing A^+^-C pair and intramolecular complementary bases, experimented in normal TMSDR system. Under acidic conditions, the HS toehold remains closed by complement bases, preventing TMSDR and signal generation. Under neutral or alkaline conditions, dissociation of A^+^-C pair destabilizes the stem, exposing the toehold and enabling TMSD activation (Fig. 3H). Fluorescence kinetics experiments demonstrated significantly higher signals at pH 8.0 compared to pH 5.0 (Fig. 3I). pH-dependent studies confirmed the alkaline preference of this system (Fig. 3J and Supplementary Fig. S42), with pH^T^ near neutral conditions. Satisfactory resetability between pH 6 and 7 is observed (Fig. 3K). To output the signal in narrow pH, we combined HS with i-motif-forming sequence, balancing their alkaline and acidic preferences (Fig. 3L). This design produced optimal fluorescence under pH 6.5 (Fig. 3M), as excessive acidity inactivated the HS while alkalinity prevented the folding of i-motif-forming sequence. HS of different lengths produced optimal fluorescence value at different pH and resetability was experimentally validated (Fig. 3N and Supplementary Fig. S43). The optimal response to pH 6.5 was also demonstrated by CD, UV, and gel electrophoresis (Supplementary Fig. S44). This nucleic acid probe, with its selective response to mildly acidic pH, demonstrates significant potential for clinical applications. Thanks to the unique programmability of DNA, SHADE has both tunability and selectivity (Supplementary Table S6).

### Sensing of pH in extracellular microenvironment

**Figure 4.**
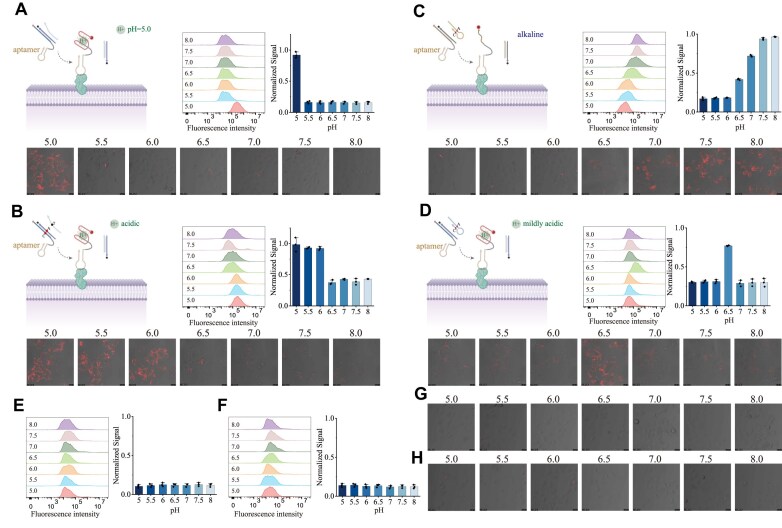
Sensing of pH in extracellular microenvironment. CLSM and flow cytometry results under different pH conditions for (**A**) SHADE, (**B**) SHADE with A^+^-C pair, (**C**) SHADE based on alkaline-preferring HS strands, and (**D**) integration of i-motif with HS. CLSM and flow cytometry results under different pH conditions after modification of (**E**,**G**) aptamer sequences and (**F**,**H**) pH-sensitive bases.

We first evaluated the biocompatibility of SHADE *in vitro* using HeLa cells. Aptamer modules were integrated for cell surface targeting, enabling specific tumor microenvironment imaging *in vivo*. While normal tissues exhibit low-level receptor expression leading to partial aptamer binding [46, 47], the lack of protons in microenvironment prevents probe activation, minimizing nonspecific signal. WST-1 assays confirmed negligible cytotoxicity (Supplementary Fig. S45). Polyacrylamide gel electrophoresis analysis indicated that SHADE components were stable in serum condition after 2-h incubation (Supplementary Fig. S46). To detect extracellular acid-driven SHADE, a c-MET-targeting aptamer was conjugated to the 3′ end of i-motif-forming sequence (Fig. 4A). Flow cytometry and CLSM revealed significantly enhanced surface fluorescence at pH 5.0. Fluorescence intensity increased under mildly acidic conditions when A^+^-C pair was introduced between i-motif-forming sequence and blocker (Fig. 4B and Supplementary Fig. S47). HS-mediated TMSDR demonstrated alkaline-preferential fluorescence imaging, with stronger signals at pH 8.0 than pH 5.0 (Fig. 4C). Combining i-motif-forming sequence with HS produced fluorescence exclusively at pH 6.5 (Fig. 4D and Supplementary Fig. S48), consistent with *in vitro* results. To validate pH-dependent membrane imaging, aptamer sequences (Fig. 4E and G) and pH-sensitive bases were randomly changed (Fig. 4F and H). Both modifications resulted in weak membrane fluorescence regardless of extracellular pH, confirming the ability of SHADE for precise pH sensing in extracellular microenvironments.

**Figure 5:**
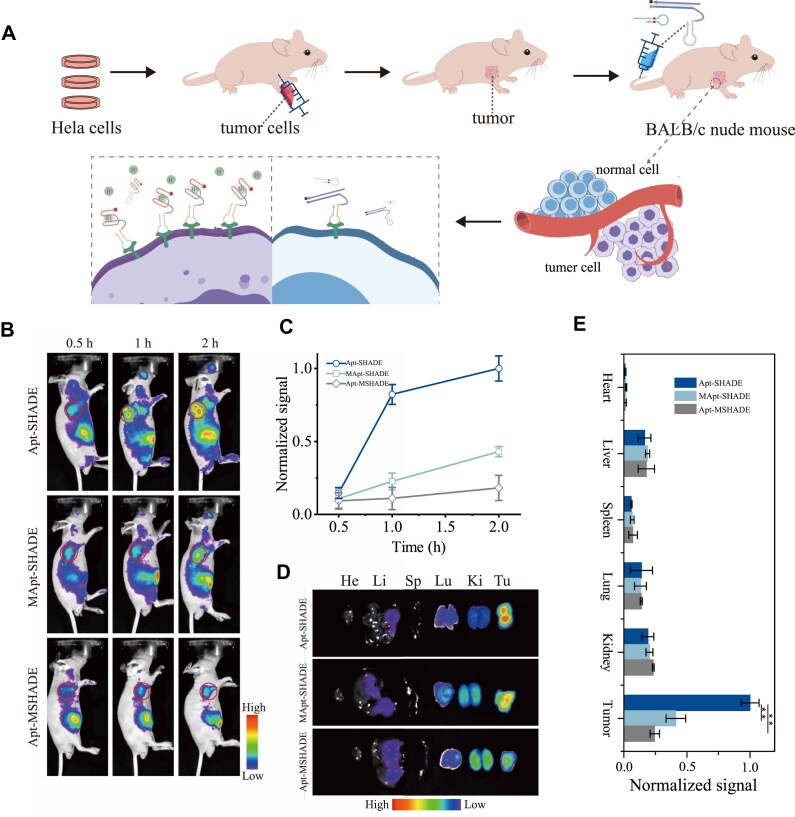
(**A**) The process of imaging *in vivo*. (**B**) Representative whole-body fluorescence images of tumor-bearing mice i.v. injected with different probes. Tumors are indicated by red circles. (**C**) Fluorescence intensity ratio between tumor and normal tissues (T/N ratio) as a function of time after different treatments (*n* = 3). (**D**) Fluorescence images of *ex vivo* organs and tumors harvested at 2 h after i.v. injection. He, Li, Sp, Lu, Ki, and Tu stand for heart, liver, spleen, lung, kidney, and tumor, respectively. (**E**) Fluorescence intensity obtained from the *ex vivo* organs and tumors in panel (D). The Cy5-labeled probe is Apt-SHADE. Apt-MSHADE is that the bases of pH response were changed, and MApt-SHADE is that bases of aptamer were changed.

To evaluate selectivity and imaging capability in solid tumors, we conducted *in vivo* fluorescence imaging experiments. Tumor xenograft model was established by subcutaneously injecting c-Met-positive HeLa cells into BALB/c nude mice, followed by intravenous administration of Cy5-labeled DNA sequences (Apt-SHADE) (Fig. 5A). Tumor fluorescence intensity gradually increased over 2 hours post-injection (Fig. 5B and C). Control experiments with changed pH-sensitive bases (Apt-MSHADE) showed minimal fluorescence due to impaired mildly acidic TME detection. Another control group with modified aptamer sequences (MApt-SHADE) had minimal fluorescence contrast increase in the tumor over the period due to its inability to specifically immobilize on tumor cell membrane. Two hours after treatment, *ex vivo* imaging of tumors and major organs revealed significantly higher fluorescence signals in Apt-SHADE-treated tumors compared to controls (Fig. 5D and E). These results demonstrated SHADE with aptamers enables simultaneous tumor cell surface and extracellular environment detection, providing more accurate imaging capabilities.

## Conclusion

The development of molecular biosensing strategies with tunable dynamic ranges is crucial for understanding and controlling critical biological processes. Real-time pH monitoring in biological media represents a particularly promising application for such systems. However, pH-responsive strategies face two major limitations: (i) restricted tunability of response ranges. Many systems operate within fixed pH windows and (ii) insufficient pH selectivity. Systems respond indiscriminately to physiological and pathological acidity, similar to diodes. The unique programmability of DNA, particularly through TMSDR, offers a powerful approach for constructing dynamic molecular systems. While numerous TMSDR-based regulatory strategies have been developed, pH response range modulation through TMSDR remains unexplored. Inspired by nature, we designed shadow strands of i-motif-forming sequence, functioning as molecular chaperones. The SHADE strategy enables pH response range tuning through shadow strand-driven TMSDR, facilitating conformational changes under acidic or alkaline conditions and allowing the design of narrow-range pH sensors.

Extensive *in vitro* and *in vivo* characterizations were performed. Orthogonal experiments with varying shadow strand lengths revealed pH sensing relationships consistent with conventional TMSDR principles. Precise pH response tuning was achieved through A^+^-C pair and HS designs. Although A and C can potentially form reverse wobble or reverse Hoogsteen pairs when deprotonated, the thermodynamic stability is weaker than protonated A^+^-C pair or the complementary base pairing in TMSDR. When combined with aptamer, SHADE effectively distinguished different extracellular pH conditions. The strategy demonstrated excellent tumor imaging performance *in vivo*. The unique advantage of the SHADE strategy reported herein is the capability to tune the response range and response in narrow pH range. This capability for accurate targeting of tumor microenvironments holds significant promise for precision medicine and could be applied to diagnose various pH-related pathologies, including inflammation, ischemia, infection, renal failure, and chronic lung disease [48, 49]. The controllable pH-responsive properties could enable numerous applications, including gene expression regulation, DNA machines, hydrogel formation, and nanoparticle assembly. Combined with other drug delivery technologies, it can achieve precise drug delivery. Furthermore, its potential combination with other molecular sensors could enable tracking of complex molecular phenomena, opening new possibilities for DNA nanostructure applications in whole organisms.

## Supplementary Material

gkaf849_Supplemental_File

## Data Availability

All data supporting the findings of this study are available within the article and its supplementary information or will be made available from the authors upon request.

## Supplementary data

Supplementary data is available at NAR online.
