# Supplementary material for: Rational design of tunable pH switches through shadow-strand hybridization-actuated displacement engineering
Source: Nucleic Acids Res. 2025 Sep 5;53(17):gkaf849. doi: 10.1093/nar/gkaf849 (PMC12412784; doi:10.1093/nar/gkaf849)
Supplement: gkaf849_Supplemental_File [file gkaf849_supplemental_file.pdf]

Supporting Information

Rational design of tunable pH switches through shadow-strand hybridization-actuated displacement engineering

Xiaole Han[1,#], Xiangyu Dong[1,#], Xiaomei Lin [1,#],Hongyan Yu[1,#], Li Zhang[1], Weitao Wang[1],Yaoyi Zhang[1], Jianbo Jiang[1], Xingyu Liu[†], Gang Yang[*,2], Yongcan Guo[*,3],and Guoming Xie[*,1]

[1]Key Laboratory of Clinical Laboratory Diagnostics (Chinese Ministry of Education), College of Laboratory Medicine, Chongqing Medical University, Chongqing, 400016, PR China.

[2]Department of Neurosurgery, The First Affiliated Hospital of Chongqing Medical University, Chongqing 400016, PR China

[3]Clinical Laboratory of Traditional Chinese Medicine Hospital Affiliated to Southwest Medical University, LuZhou Key Laboratory of Nanobiosensing and Microfluidic Point-of-Care Testing, Luzhou 646000, P. R. China

[*] To whom correspondence should be addressed. Tel: +86 23 68485240; Fax: +86 23 68485239; Email: guomingxie@cqmu.edu.cn

Correspondence may also be addressed to Gang Yang. Email: gangyang@hospital.cqmu.edu.cn and Yongcan Guo Email: swtcmlab@163.com

[#]The authors wish it to be known that, in their opinion, the first and second authors should be regarded as Joint First Authors.

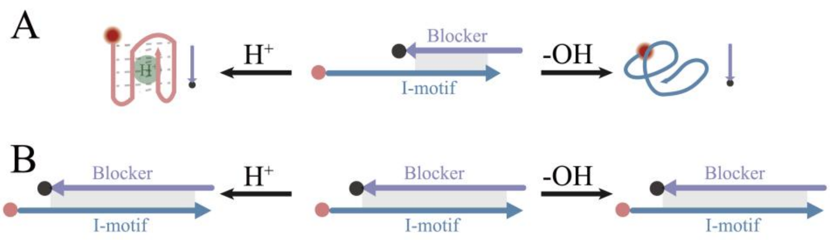

Figure S1. Schematic diagram of pH response when blocker is too short (A) and too long (B).

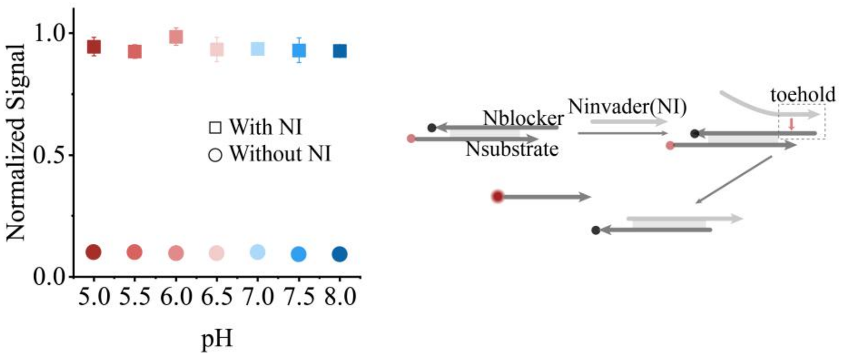

Figure S2. Effect of pH on normal strand displacement reactions. The influence of pH on TMSDR is weak.

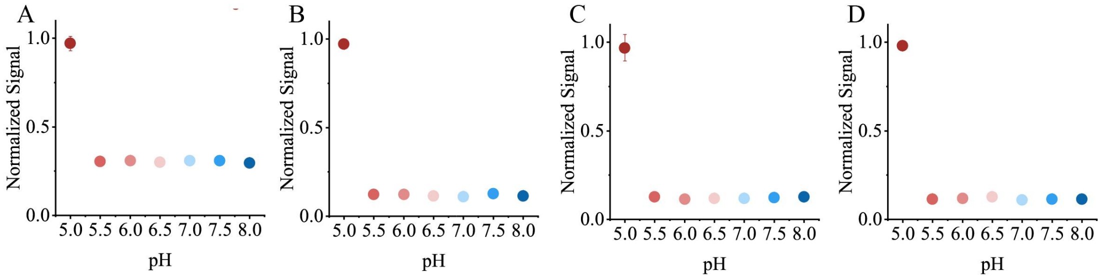

Figure S3. Different blocking ratios. Motif-to-blocker concentration ratios is (A) 1:1, (B) 1:1.5, (C) 1:2, and (D) 1:2.5.

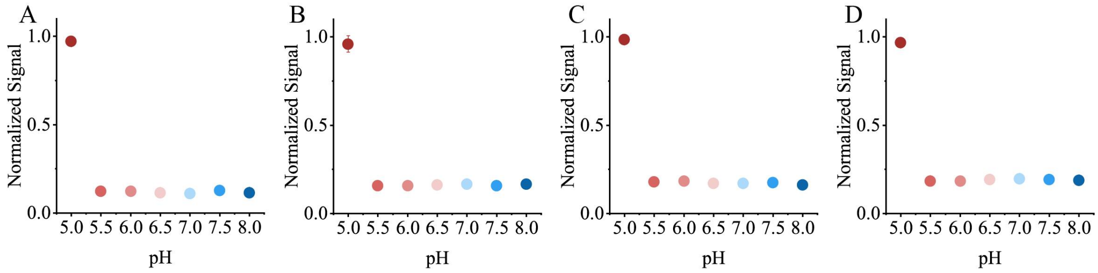

Figure S4. Different shadow strand concentrations: (A) 150 nM, (B) 200 nM, (C) 250 nM, and (D) 300 nM.

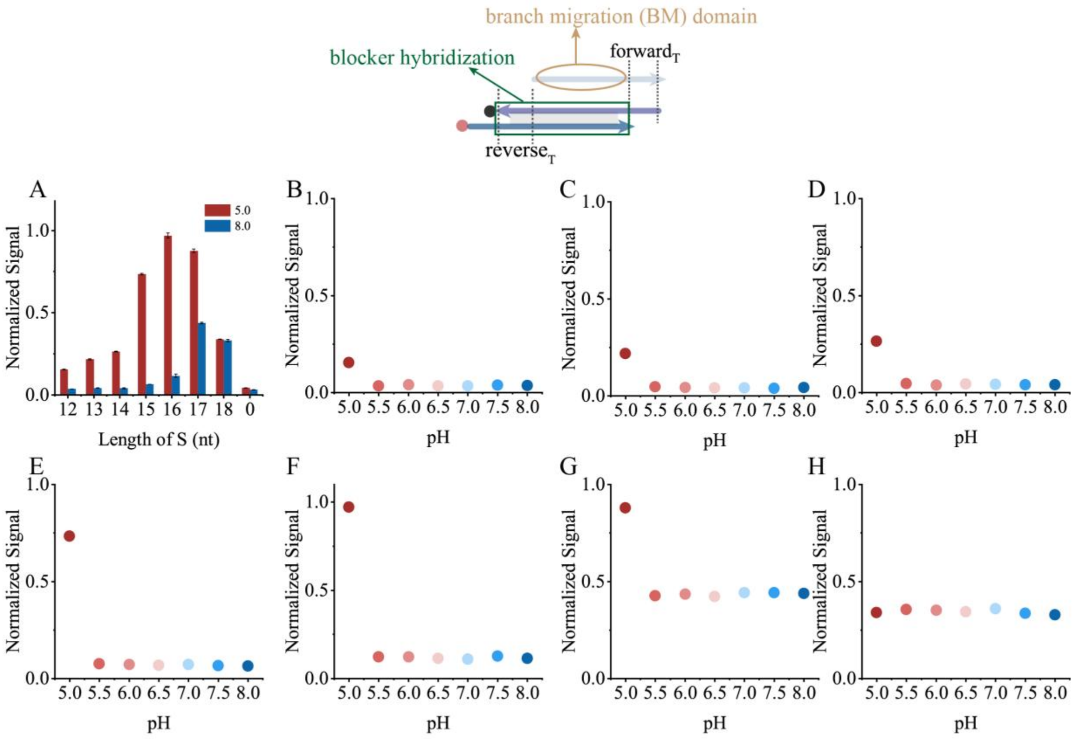

Figure S5. Branch migration domain lengths. (A) Signal comparison of pH 5.0 and pH 8.0 with different shadow lengths (forward toehold fixed at 8 nt). Branch migration domain is (B) 4 nt, (C) 5 nt, (D) 6 nt, (E) 7 nt, (F) 8 nt, (G) 9 nt, and (H) 10 nt.

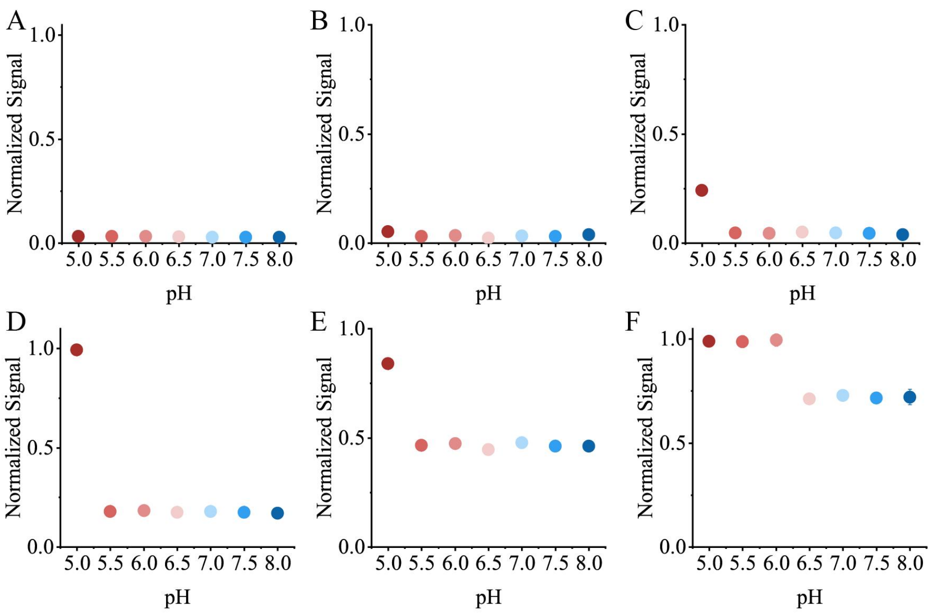

Figure S6. Different toehold lengths: (A) 0 nt, (B) 4 nt, (C) 6 nt, (D) 8 nt, (E) 10 nt, and (F) 12 nt.

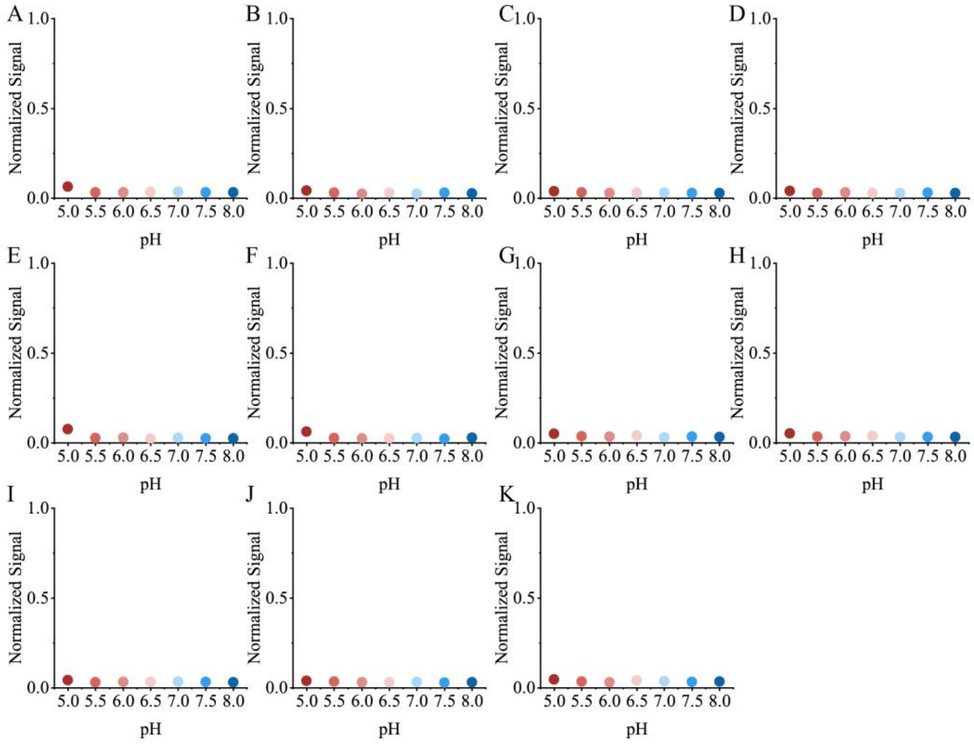

Figure S7. Signals for different blocker hybridization lengths without shadow strands. Blocker is (A) 15 nt, (B) 16 nt, (C) 17 nt, (D) 18 nt, (E) 19 nt, (F) 20 nt, (G) 21 nt, (H) 22 nt, (I) 23 nt, (J) 24 nt, and (K) 25 nt.

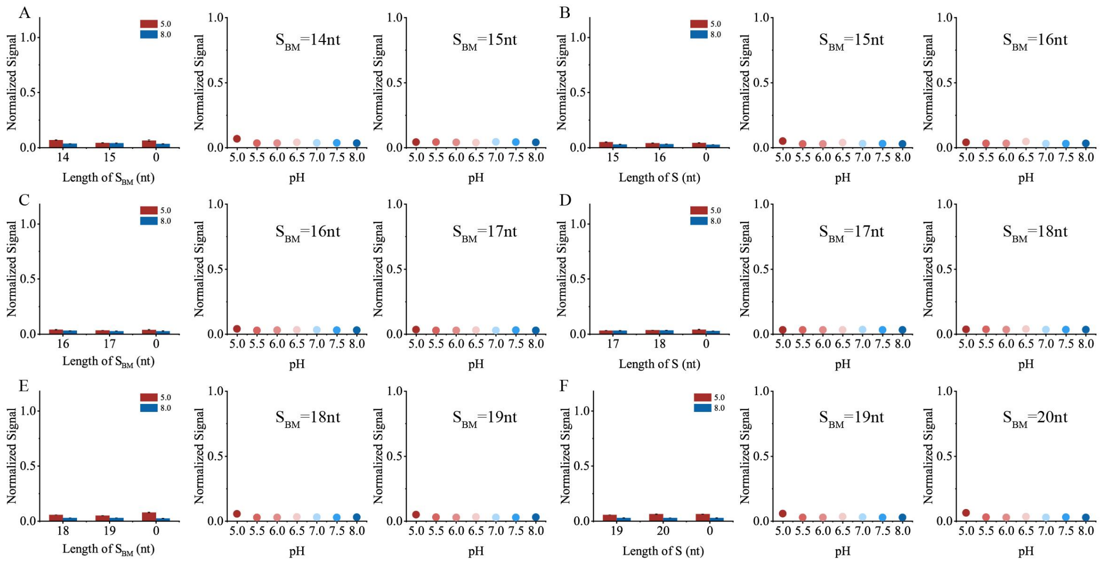

Figure S8. Forward toehold at 0 nt, blocker hybridization length is (A) 15 nt, (B) 16 nt, (C) 17 nt, (D) 18 nt, (E) 19 nt, (F) 20 nt, with varying shadow branch migration lengths.

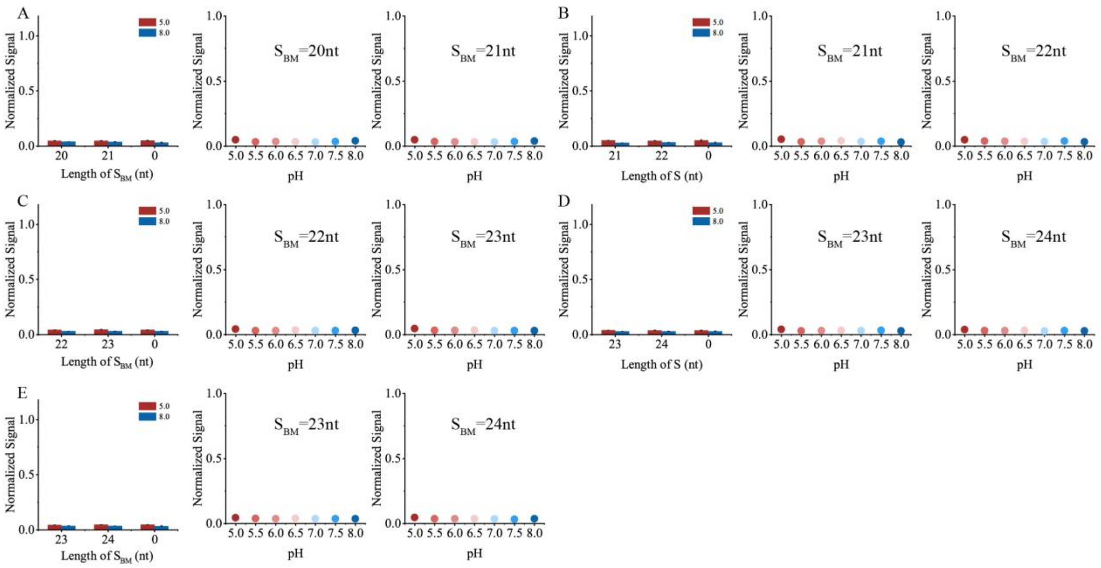

Figure S9. The fluorescence signal of different lengths of blockers and shadows with 0 nt toehold. Blocker hybridization length is (A) 21 nt, (B) 22 nt, (C) 23 nt, (D) 24 nt, (E) 25 nt, with varying shadow branch migration lengths.

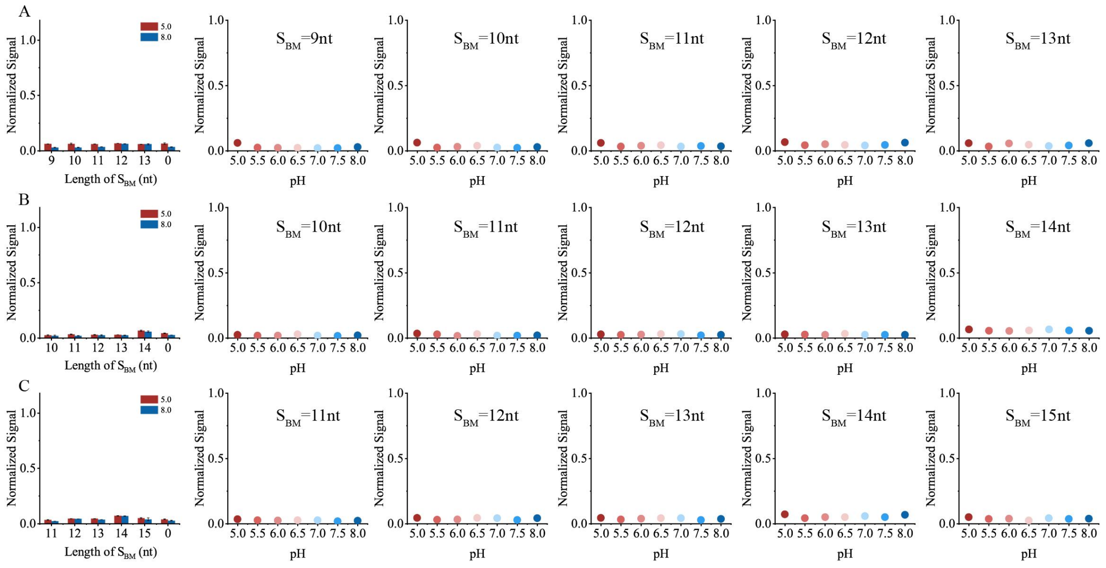

Figure S10. The fluorescence signal of different lengths of blockers and shadows with 4 nt toehold. Blocker hybridization length is (A) 15 nt, (B) 16 nt, (C) 17 nt, with varying shadow branch migration lengths.

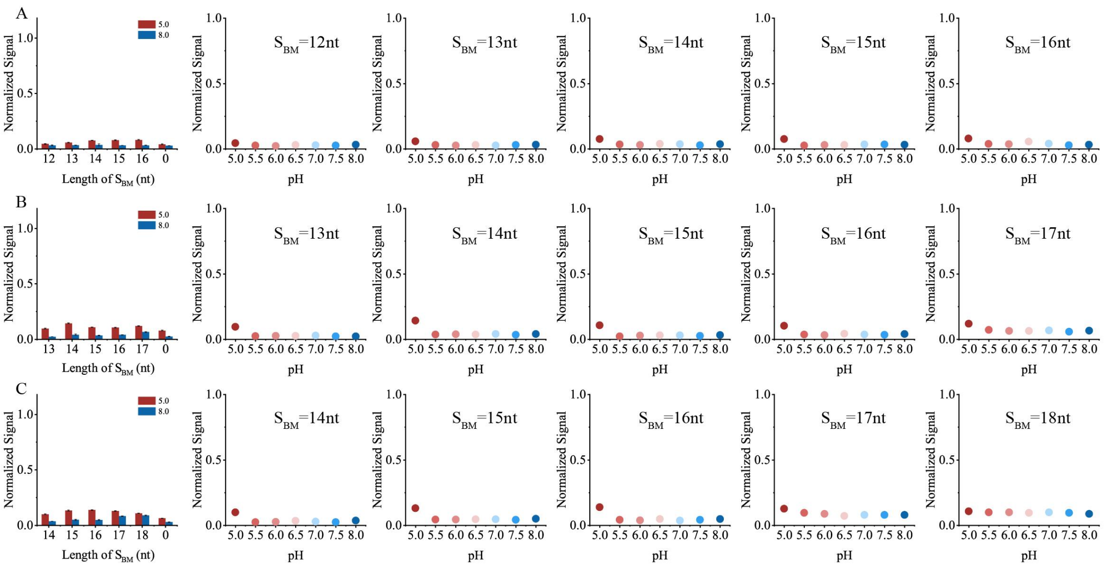

Figure S11. The fluorescence signal of different lengths of blockers and shadows with 4 nt toehold. Blocker hybridization length is (A) 18 nt, (B) 19 nt, (C) 20 nt, with varying shadow branch migration lengths.

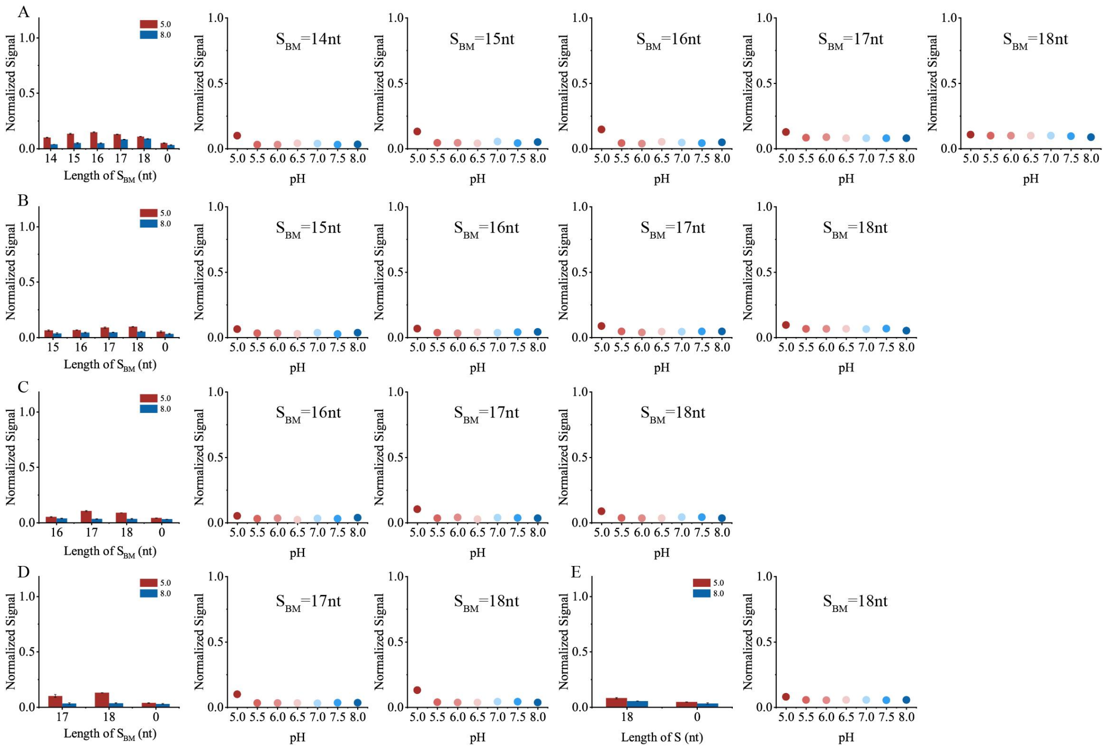

Figure S12. The fluorescence signal of different lengths of blockers and shadows with 4 nt toehold. Blocker hybridization length is (A) 21 nt, (B) 22 nt, (C) 23 nt, (D) 24 nt, (E) 25 nt, with varying shadow branch migration lengths.

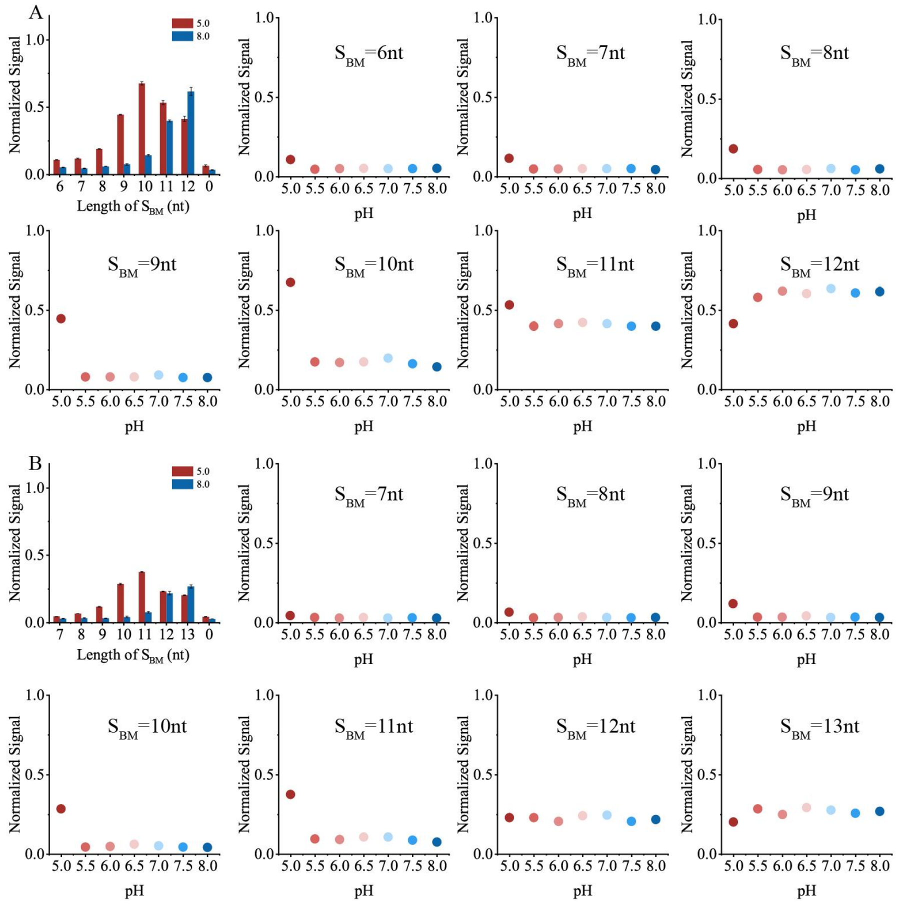

Figure S13. The fluorescence signal of different lengths of blockers and shadows with 6 nt toehold. Blocker hybridization length is (A) 15 nt, (B) 16 nt, with varying shadow branch migration lengths.

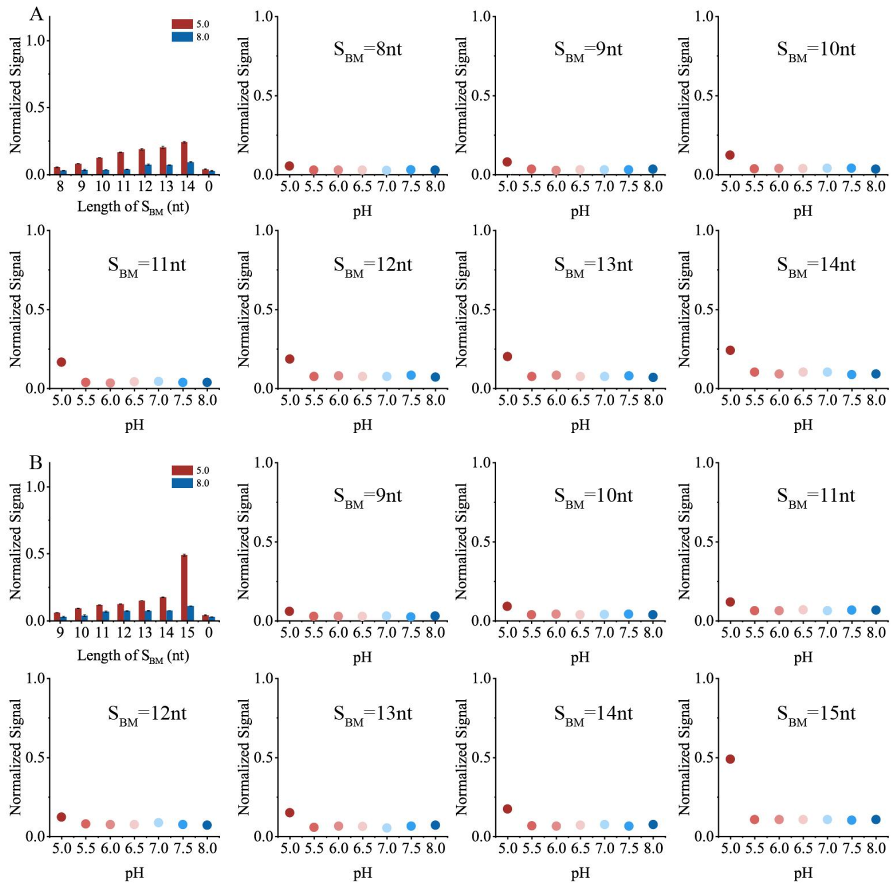

Figure S14. The fluorescence signal of different lengths of blockers and shadows with 6 nt toehold. Blocker hybridization length is (A) 17 nt, (B) 18 nt, with varying shadow branch migration lengths.

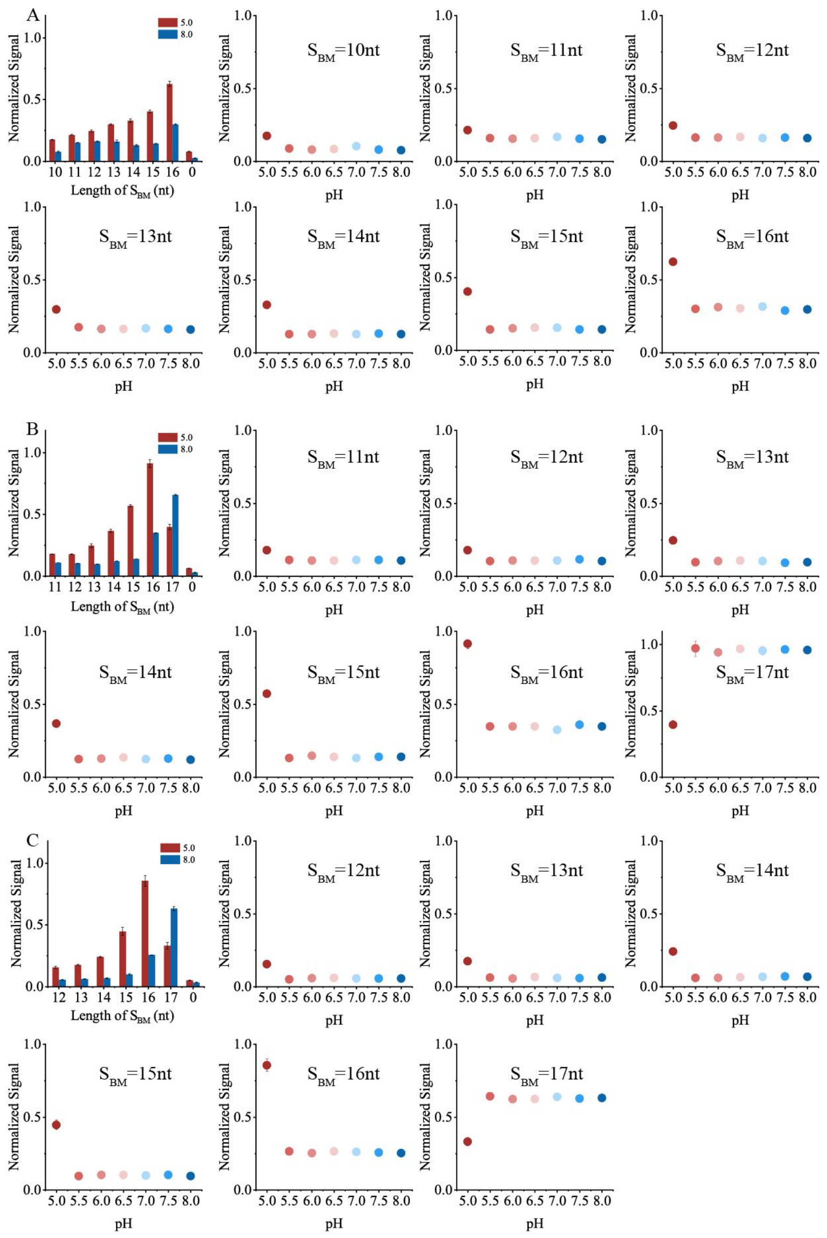

Figure S15. The fluorescence signal of different lengths of blockers and shadows with 6 nt toehold. Blocker hybridization length is (A) 19 nt, (B) 20 nt, (C) 21 nt, with varying shadow branch migration lengths.

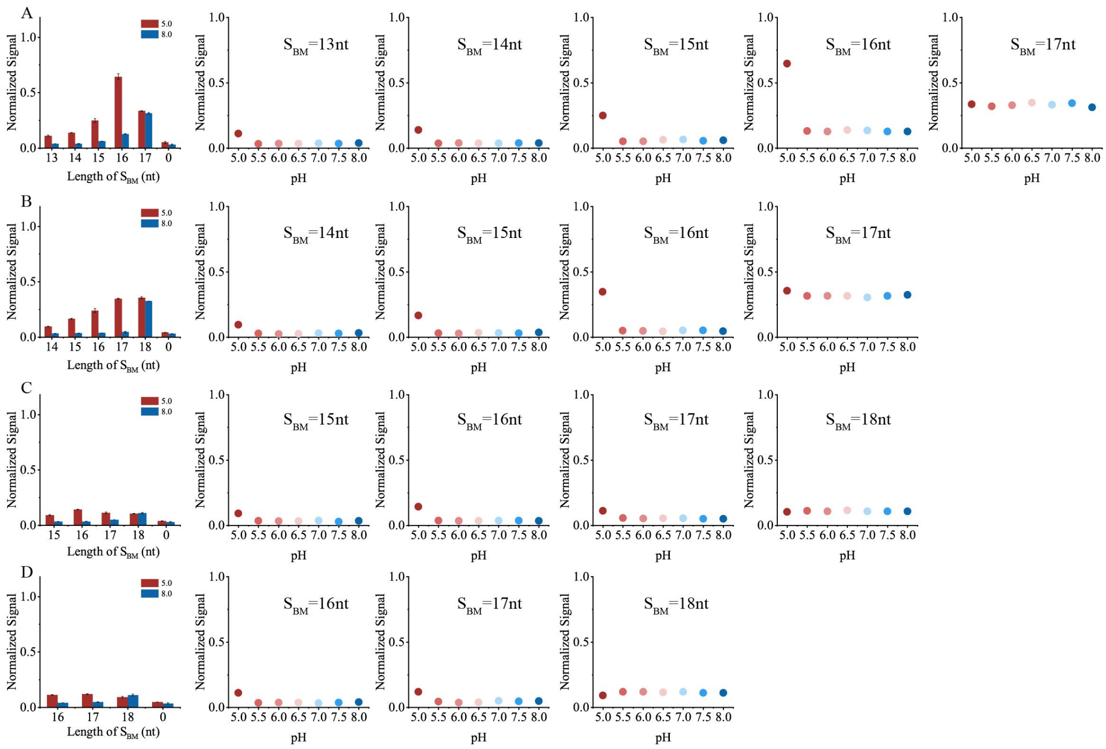

Figure S16. The fluorescence signal of different lengths of blockers and shadows with 6 nt toehold. Blocker hybridization length is (A) 22 nt, (B) 23 nt, (C) 24 nt, (D) 25 nt, with varying shadow branch migration lengths.

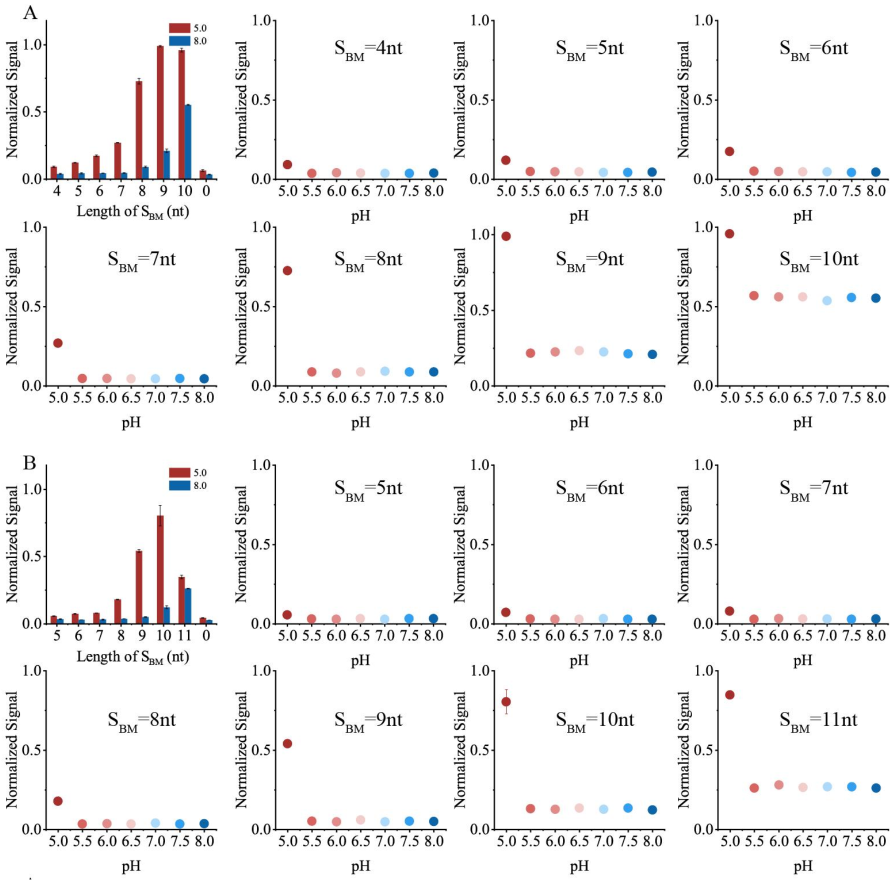

Figure S17. The fluorescence signal of different lengths of blockers and shadows with 8 nt toehold. Blocker hybridization length is (A) 15 nt, (B) 16 nt, with varying shadow branch migration lengths.

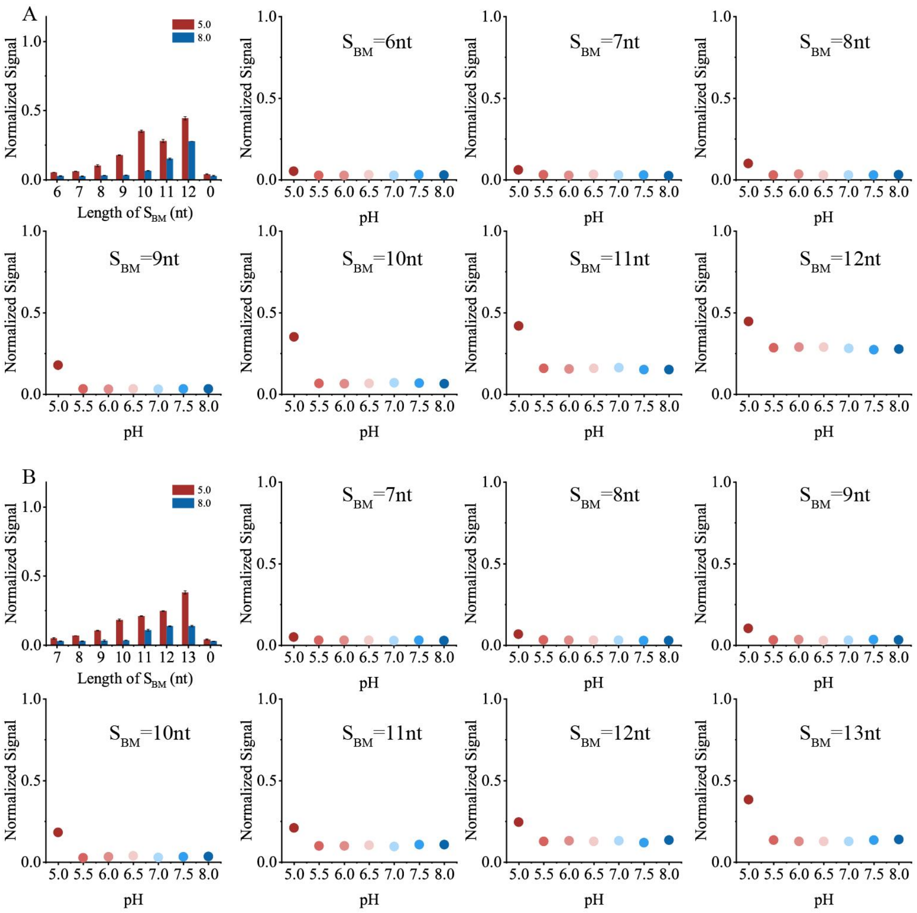

Figure S18. The fluorescence signal of different lengths of blockers and shadows with 8 nt toehold. Blocker hybridization length is (A) 17 nt, (B) 18 nt, with varying shadow branch migration lengths.

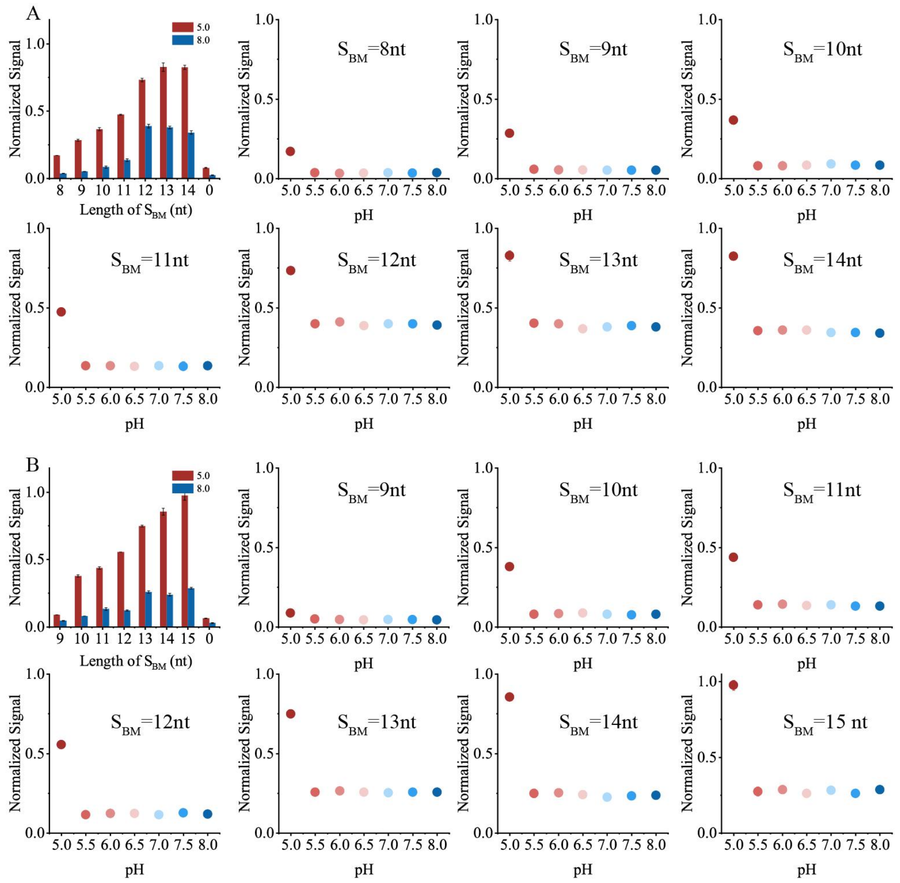

Figure S19. The fluorescence signal of different lengths of blockers and shadows with 8 nt toehold. Blocker hybridization length is (A) 19 nt, (B) 20 nt, with varying shadow branch migration lengths.

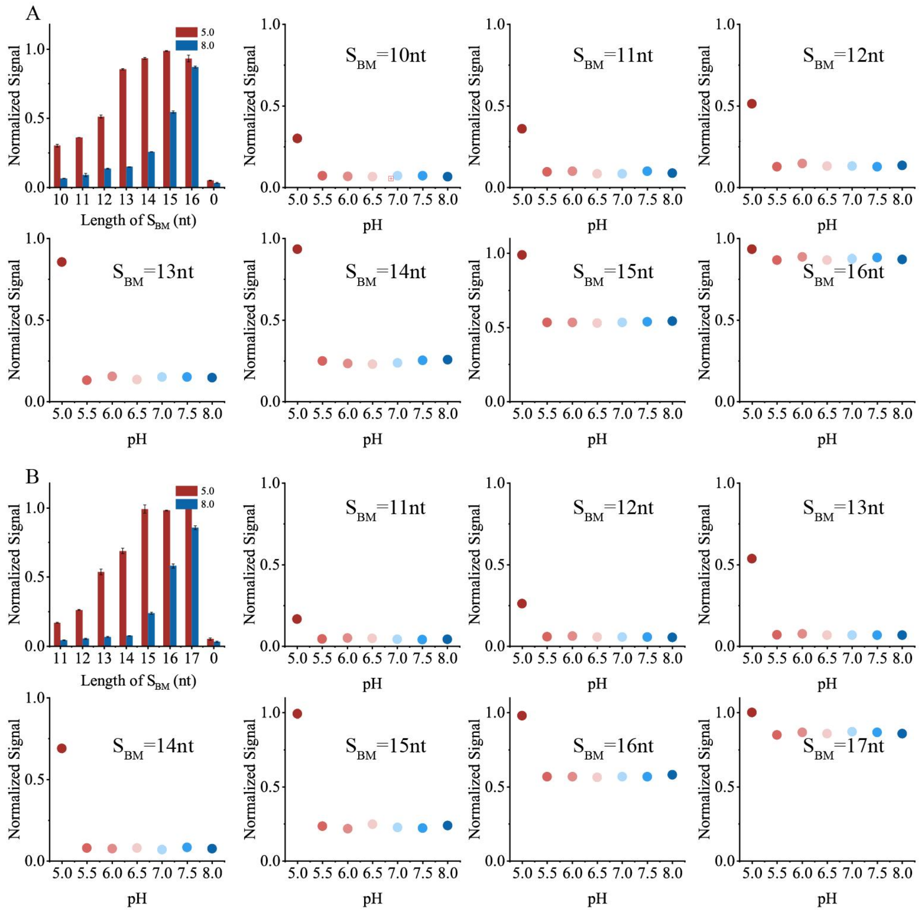

Figure S20. The fluorescence signal of different lengths of blockers and shadows with 8 nt toehold. Blocker hybridization length is (A) 21 nt, (B) 22 nt, with varying shadow branch migration lengths.

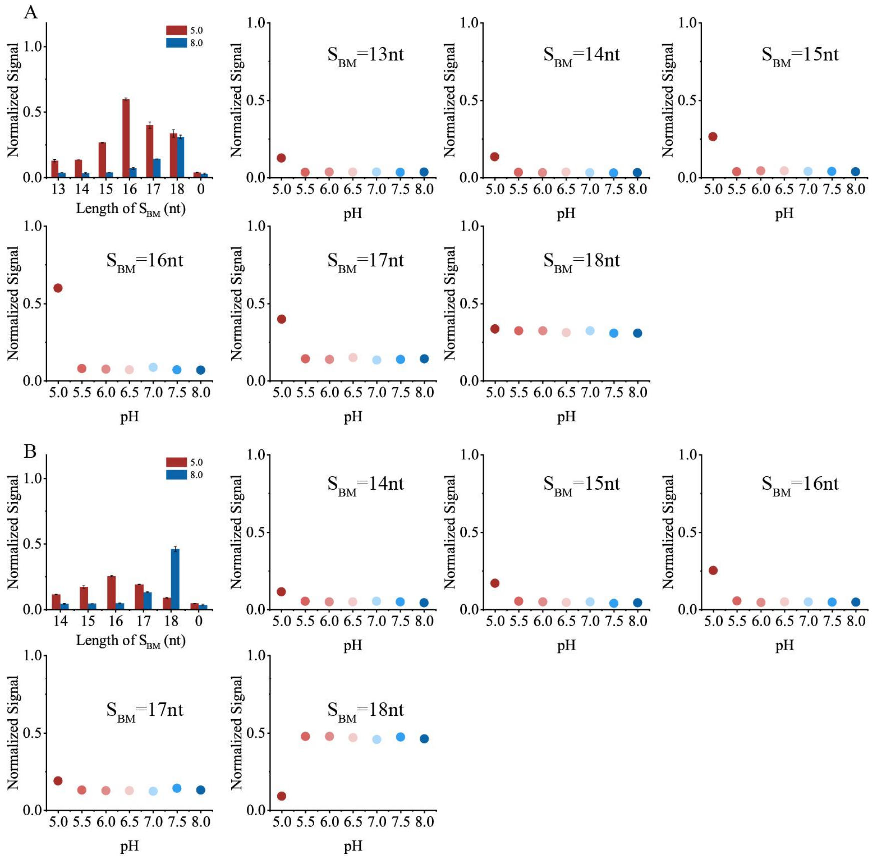

Figure S21. The fluorescence signal of different lengths of blockers and shadows with 8 nt toehold. Blocker hybridization length is (A) 24 nt, (B) 25 nt, with varying shadow branch migration lengths.

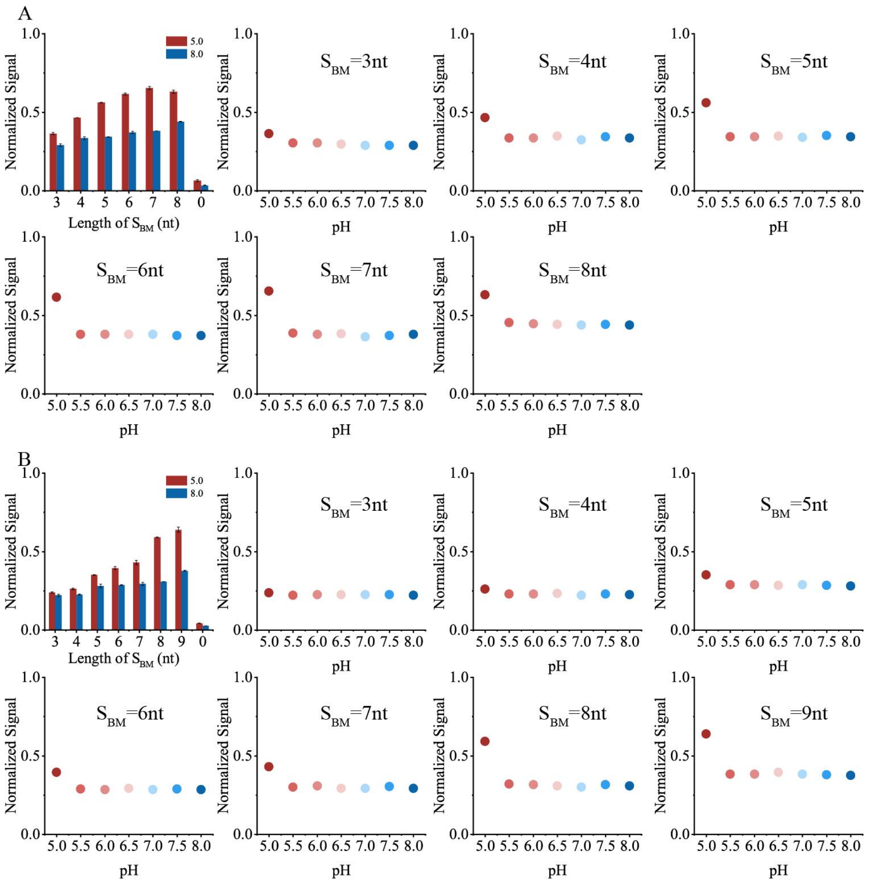

Figure S22. The fluorescence signal of different lengths of blockers and shadows with 10 nt toehold. Blocker hybridization length is (A) 15 nt, (B) 16 nt, with varying shadow branch migration lengths.

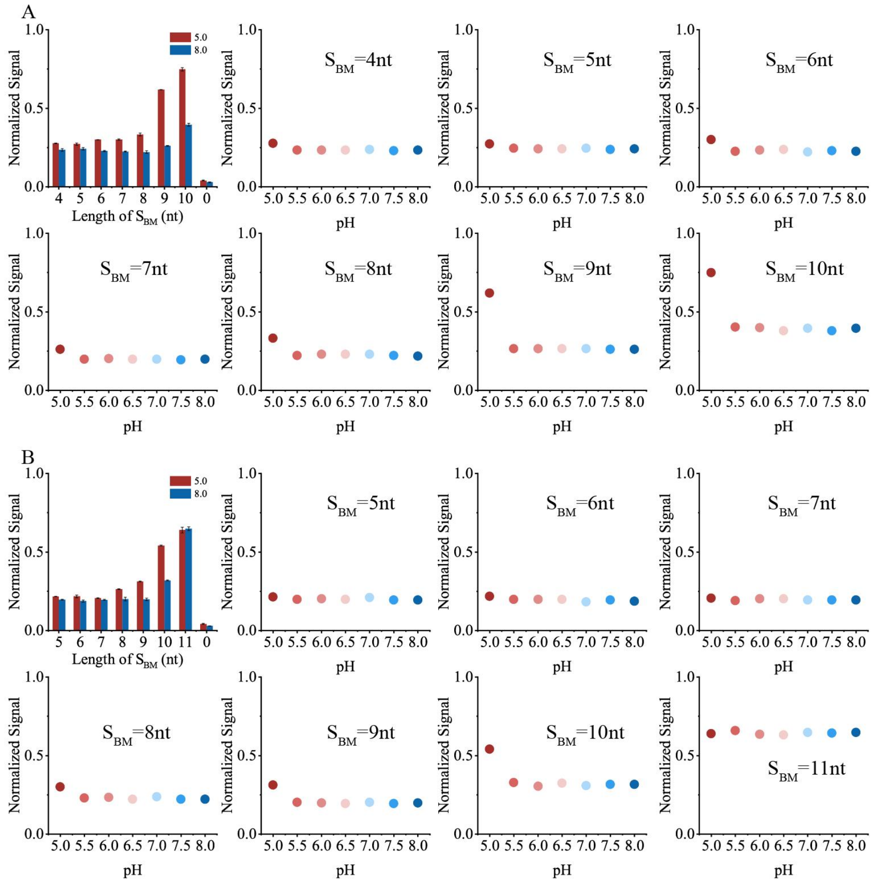

Figure S23. The fluorescence signal of different lengths of blockers and shadows with 10 nt toehold. Blocker hybridization length is (A) 17 nt, (B) 18 nt, with varying shadow branch migration lengths.

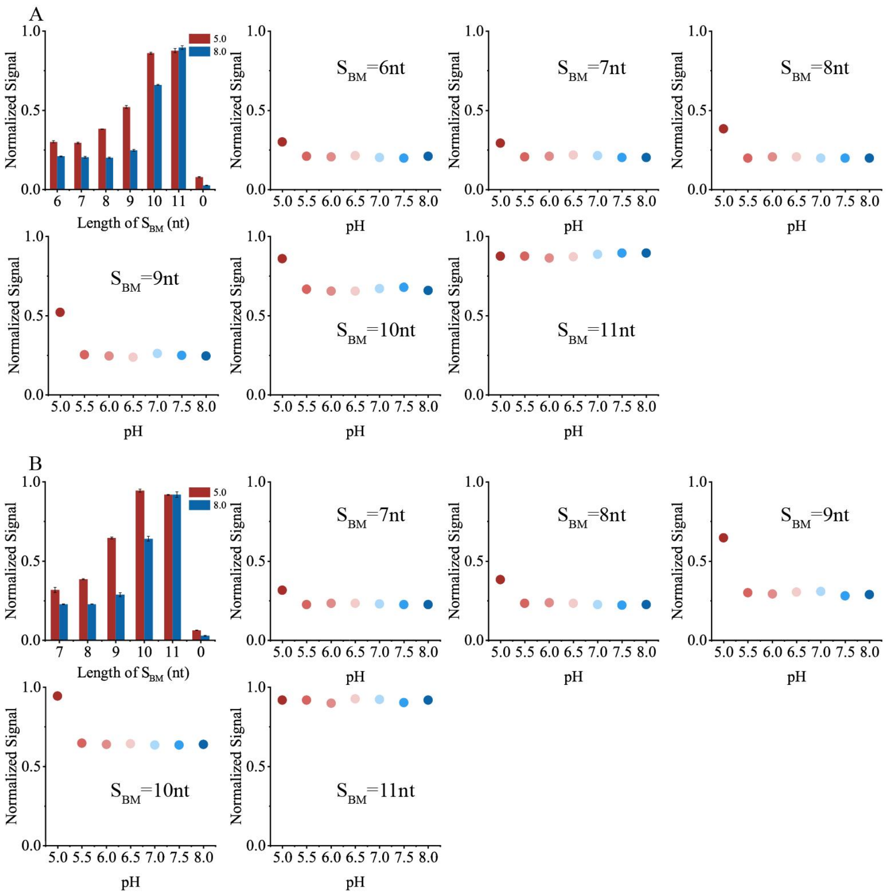

Figure S24. The fluorescence signal of different lengths of blockers and shadows with 10 nt toehold. Blocker hybridization length is (A) 19 nt, (B) 20 nt, with varying shadow branch migration lengths.

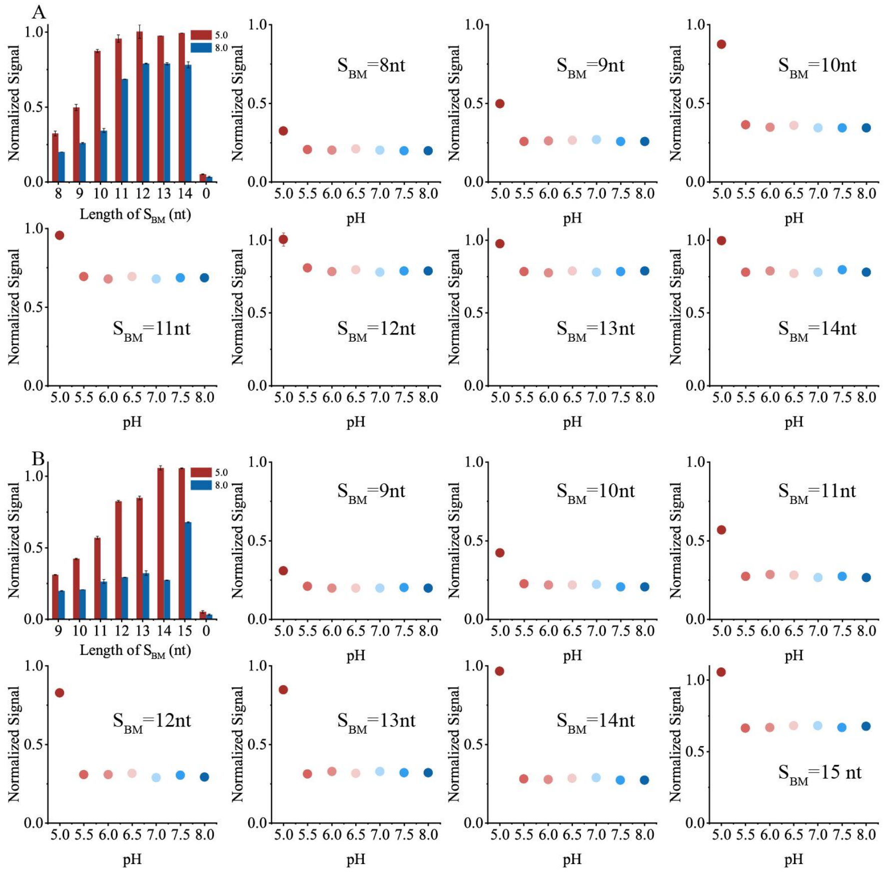

Figure S25. The fluorescence signal of different lengths of blockers and shadows with 10 nt toehold. Blocker hybridization length is (A) 21 nt, (B) 22 nt, with varying shadow branch migration lengths.

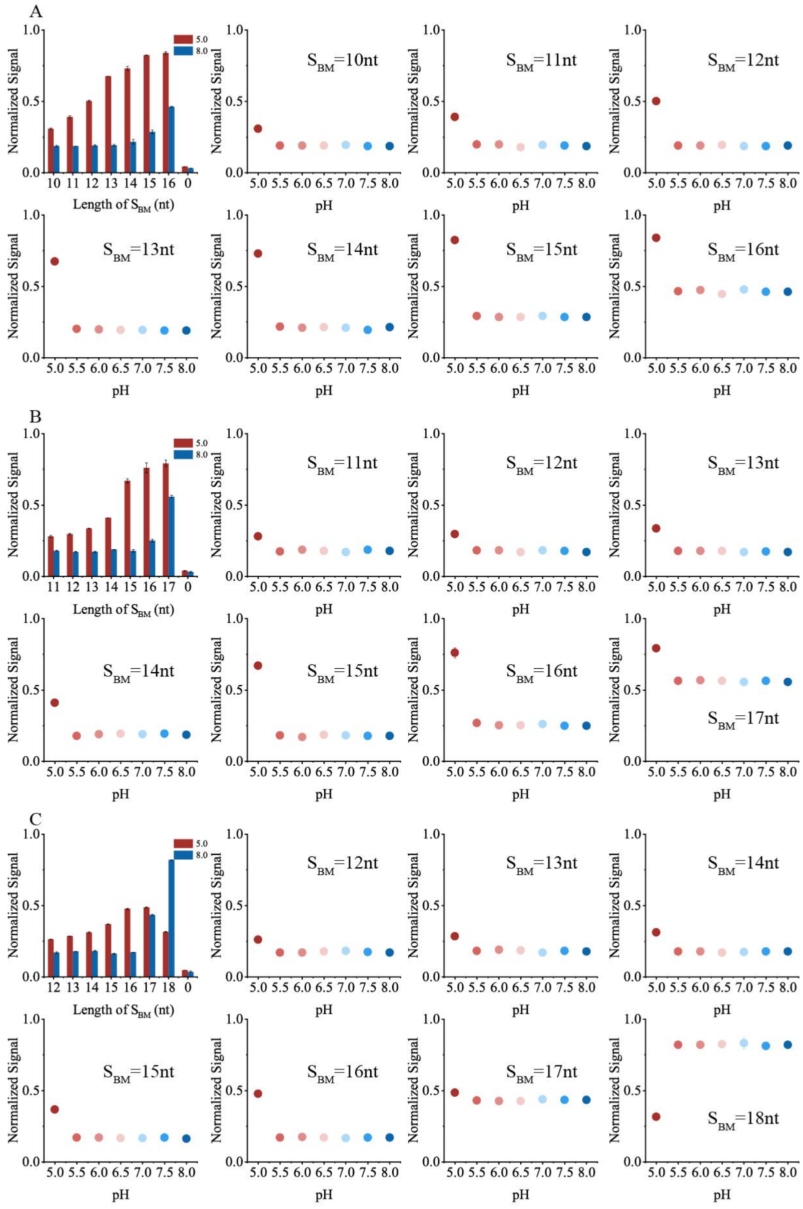

Figure S26. The fluorescence signal of different lengths of blockers and shadows with 10 nt toehold. Blocker hybridization length is (A) 23 nt, (B) 24 nt, (C) 25 nt, with varying shadow branch migration lengths.

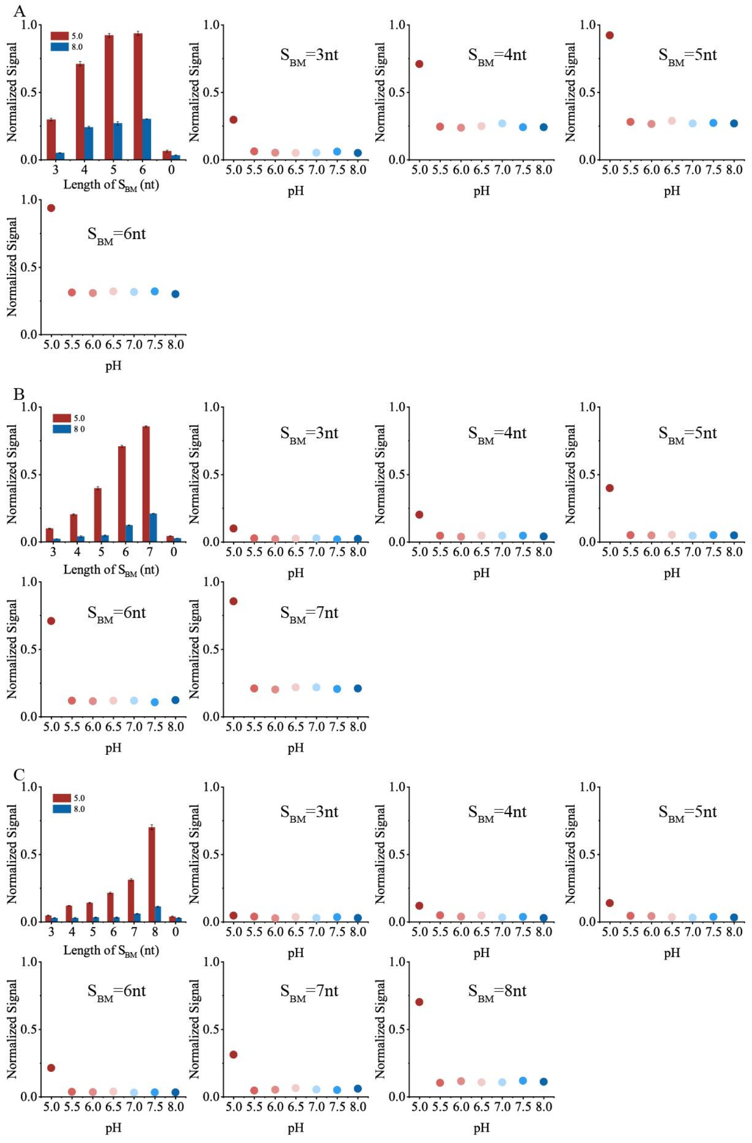

Figure S27. The fluorescence signal of different lengths of blockers and shadows with 12 nt toehold. Blocker hybridization length is (A) 15 nt, (B) 16 nt, (C) 17 nt, with varying shadow branch migration lengths.

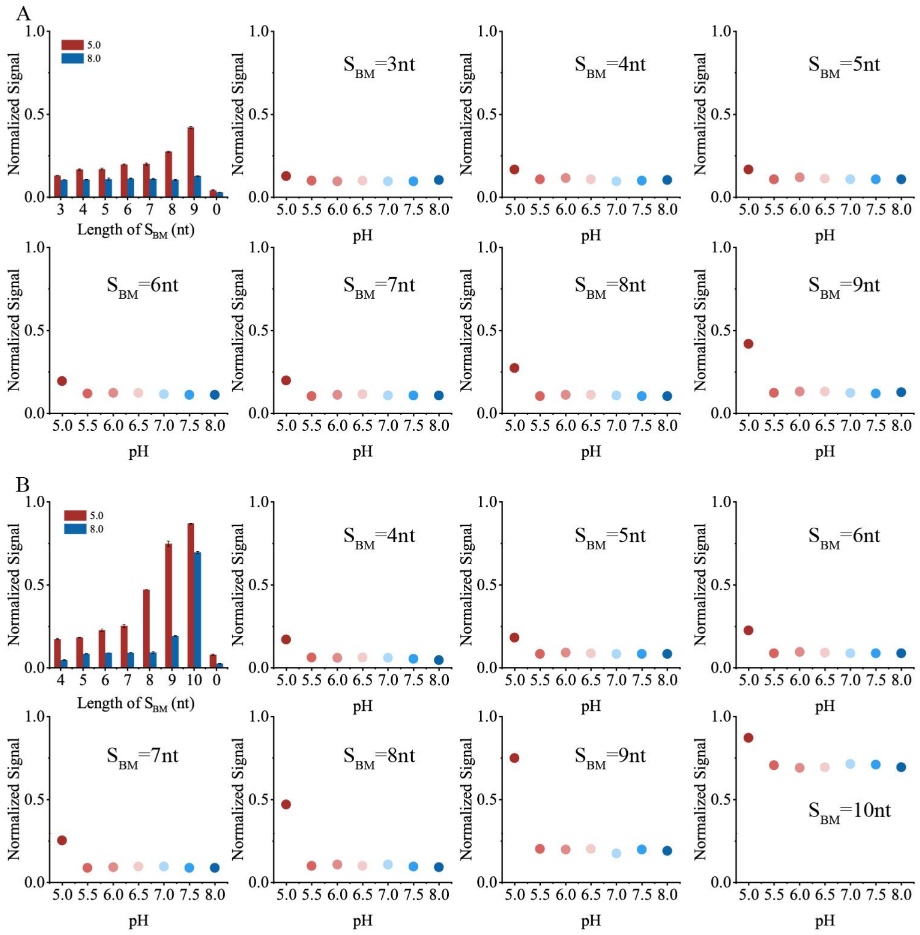

Figure S28. The fluorescence signal of different lengths of blockers and shadows with 12 nt toehold. Blocker hybridization length is (A) 18 nt, (B) 19 nt, with varying shadow branch migration lengths.

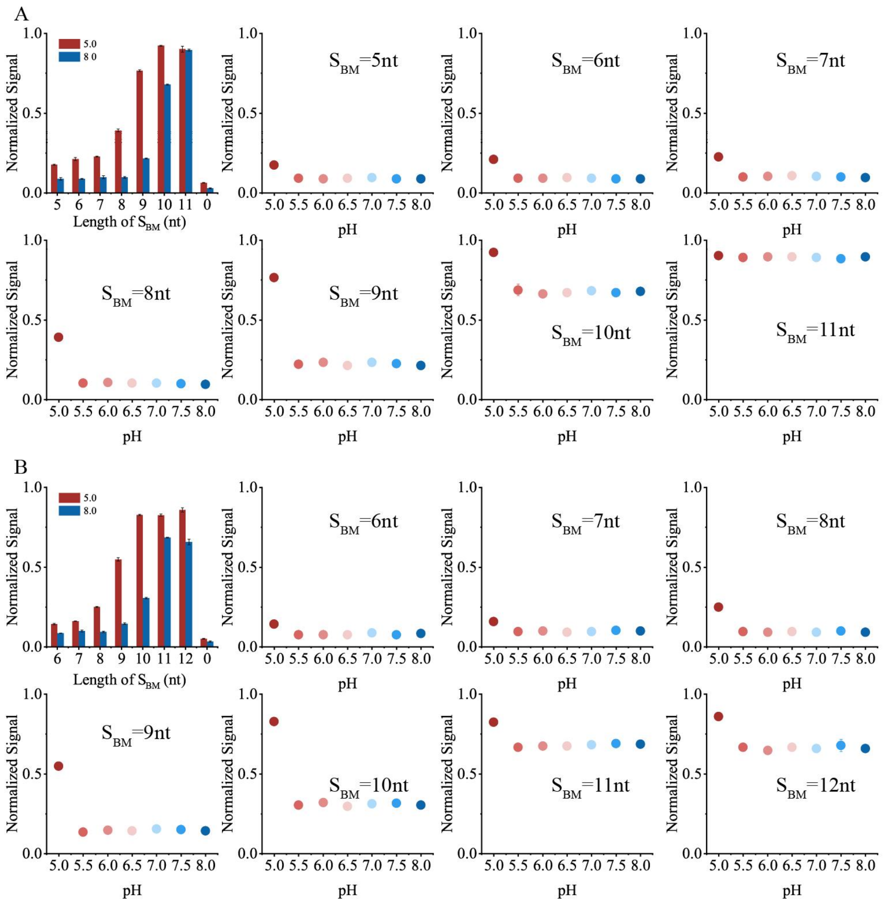

Figure S29. The fluorescence signal of different lengths of blockers and shadows with 12 nt toehold. Blocker hybridization length is (A) 20 nt, (B) 21 nt, with varying shadow branch migration lengths.

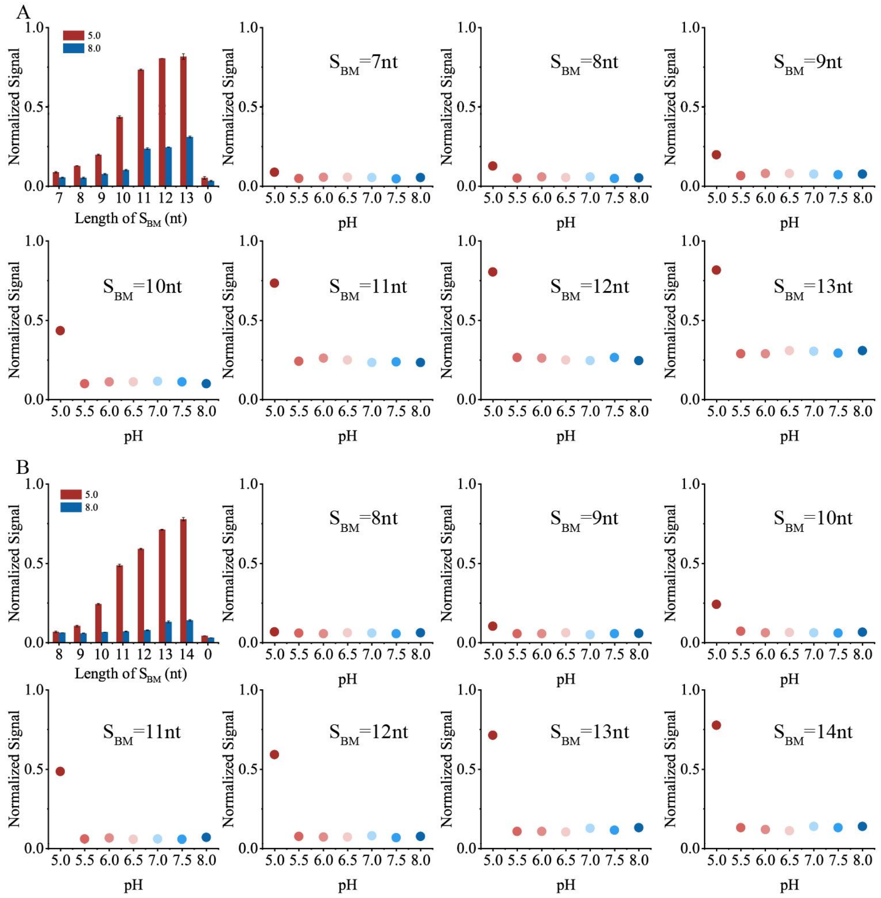

Figure S30. The fluorescence signal of different lengths of blockers and shadows with 12 nt toehold. Blocker hybridization length is (A) 22 nt, (B) 23 nt, with varying shadow branch migration lengths.

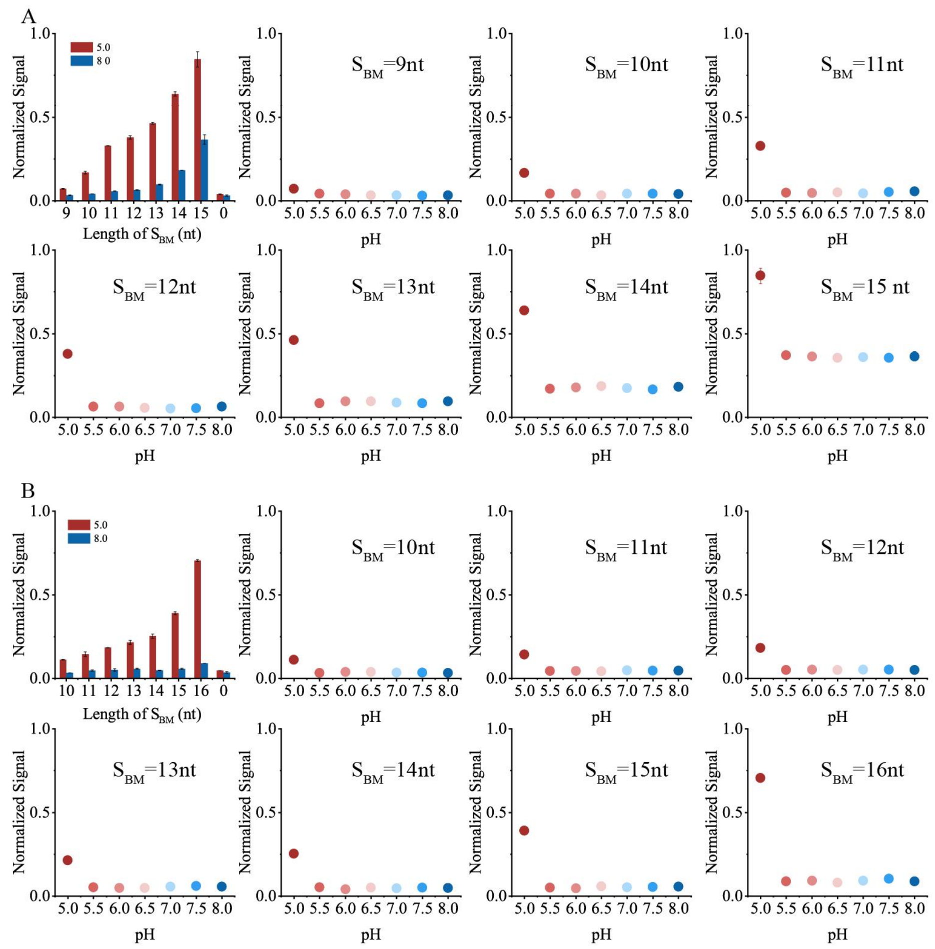

Figure S31. The fluorescence signal of different lengths of blockers and shadows with 12 nt toehold. Blocker hybridization length is (A) 24 nt, (B) 25 nt, with varying shadow branch migration lengths.

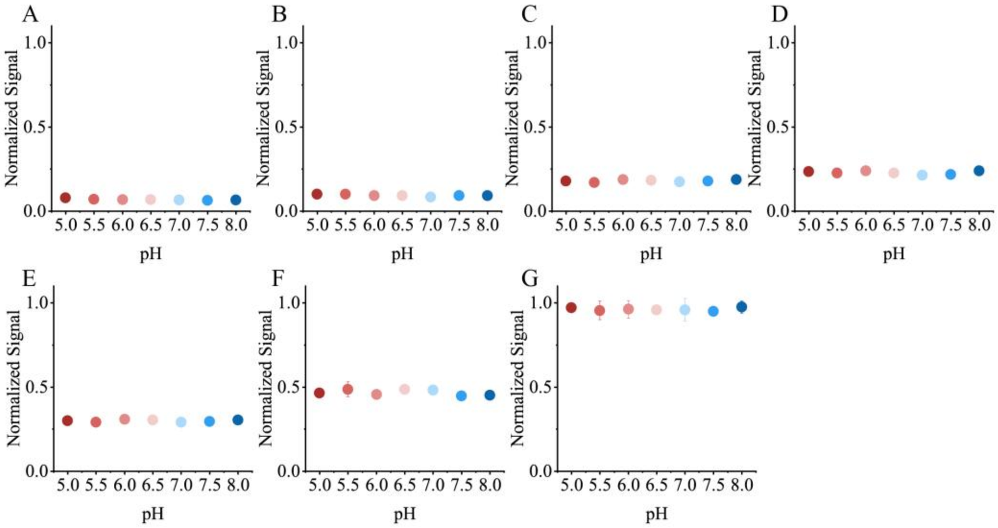

Figure S32. pH response of i-motif variants with different Δf_T-r_T: (A) -3 nt, (B) -2 nt, (C) -1 nt, (D) 0 nt, (E) 1 nt, (F) 2 nt, and (G) 3 nt.

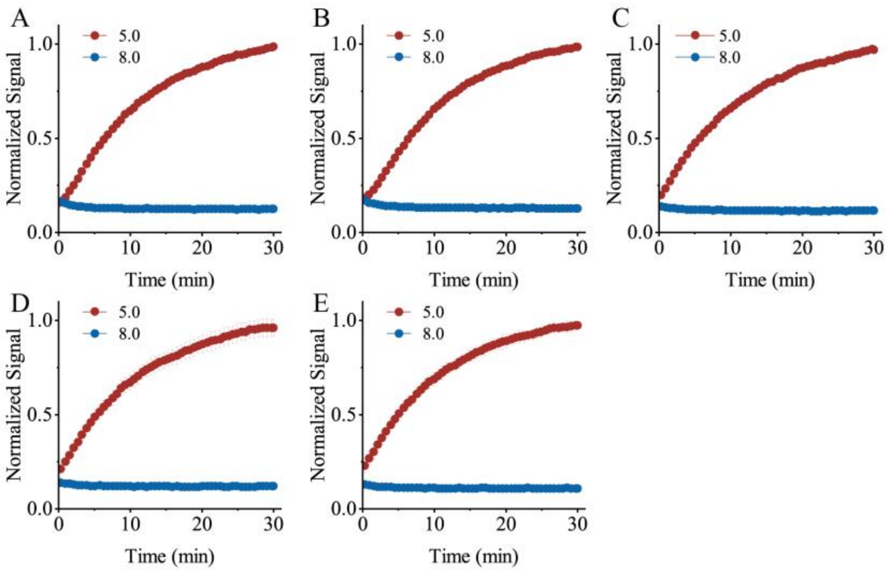

Figure S33. Shadow strand performance at different temperatures: (A) 27°C, (B) 32°C, (C) 37°C, (D) 42°C, and (E) 47°C.

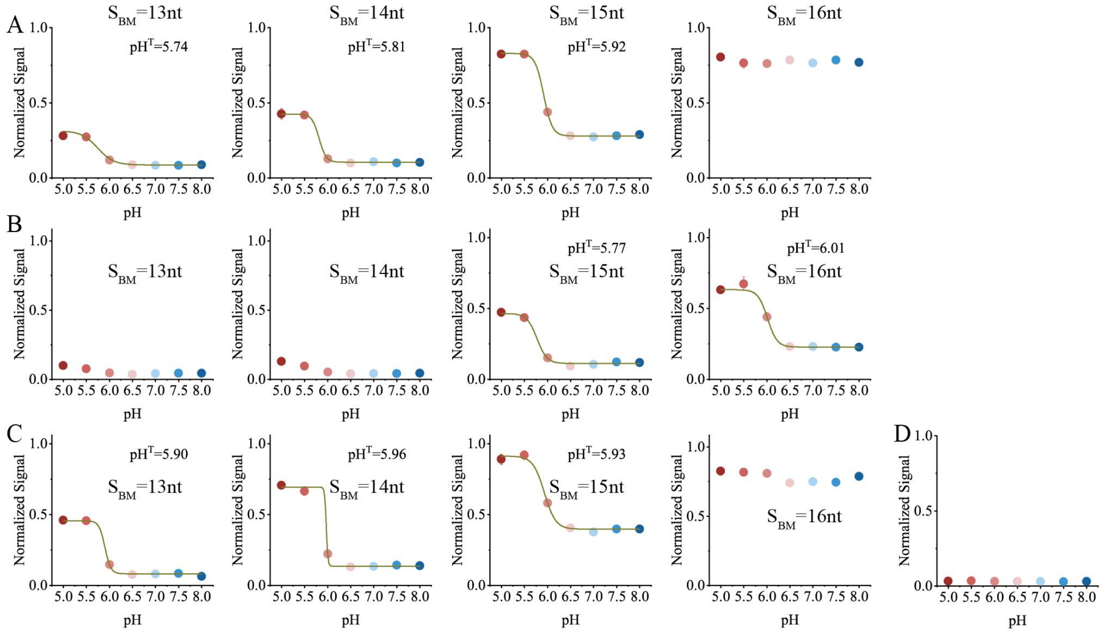

Figure S34. Introduction of one C-A pairs at the 3' end with different toehold lengths: (A) 8 nt, (B) 7 nt, (C) 9 nt. (D) Fluorescence signals at different pH values without shadow strands.

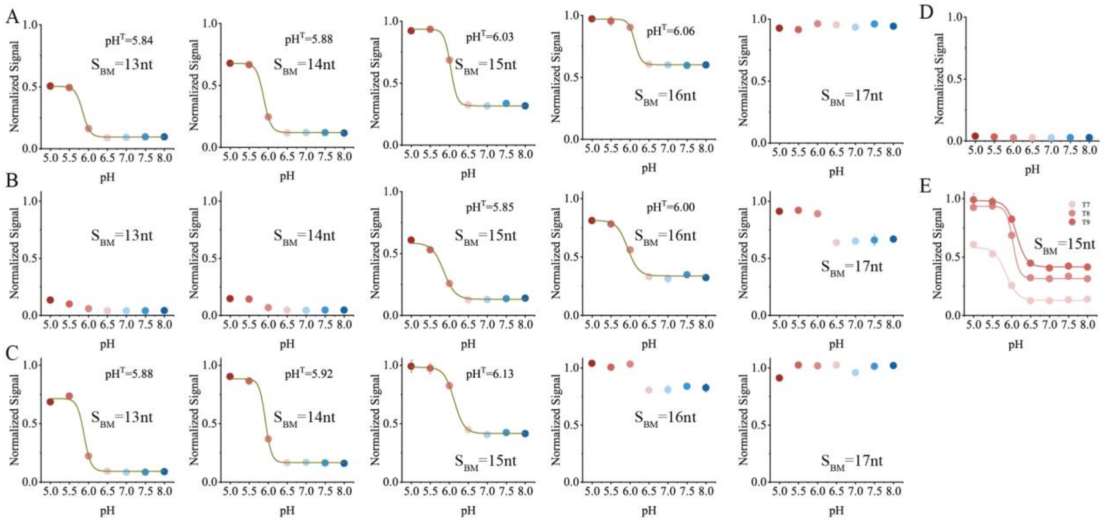

Figure S35. (A) Introduction of one C-A pairs at the 5' end with different toehold lengths: (A) 8 nt, (B) 7 nt, (C) 9 nt. (D) Fluorescence signals of different shadow toehold. (E) Fluorescence signals at different pH values without shadow strands.

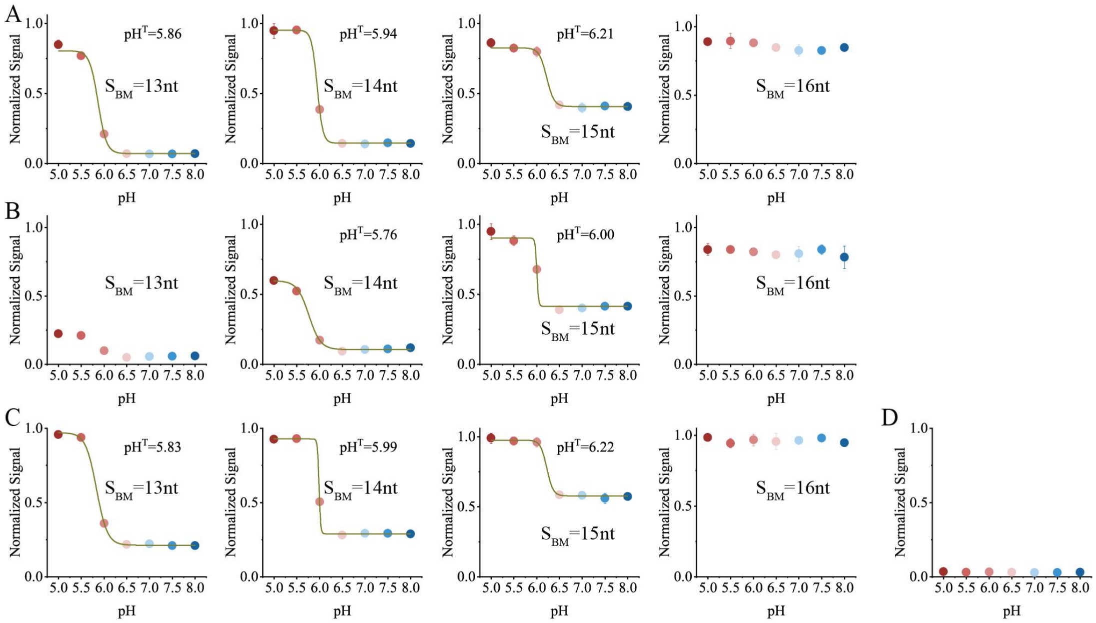

Figure S36. (A) Introduction of one C-A pairs at both the 3' and 5' ends with different toehold lengths: (A) 8 nt, (B) 7 nt, (C) 9 nt. (D) Fluorescence signals at different pH values without shadow strands.

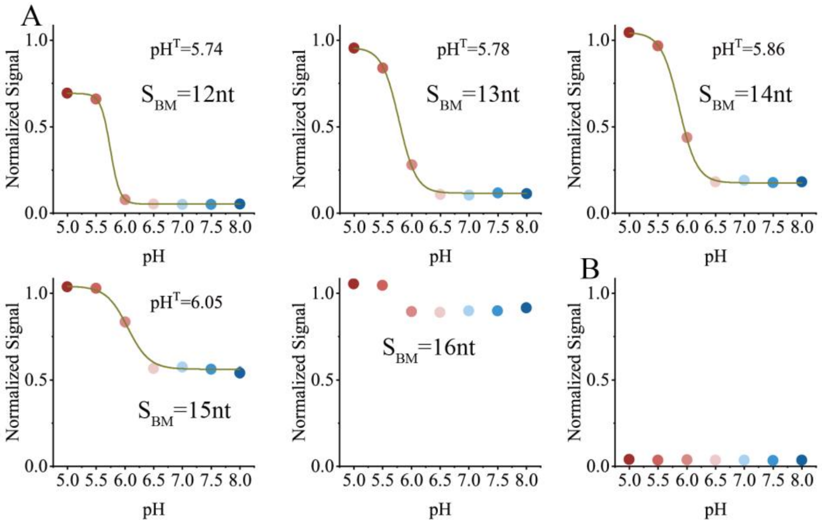

Figure S37. (A) Introduction of two C-A pairs at the 3' end with 8 nt toehold. (B) Fluorescence signals at different pH values without shadow strands.

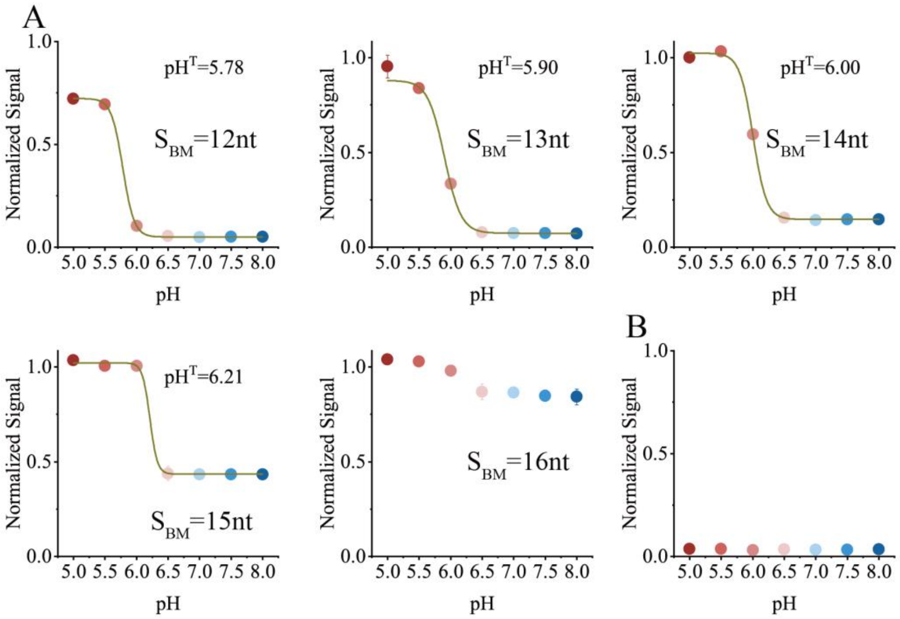

Figure S38. (A) Introduction of two C-A pairs at the 5' end with 8 nt toehold. (B) Fluorescence signals at different pH values without shadow strands.

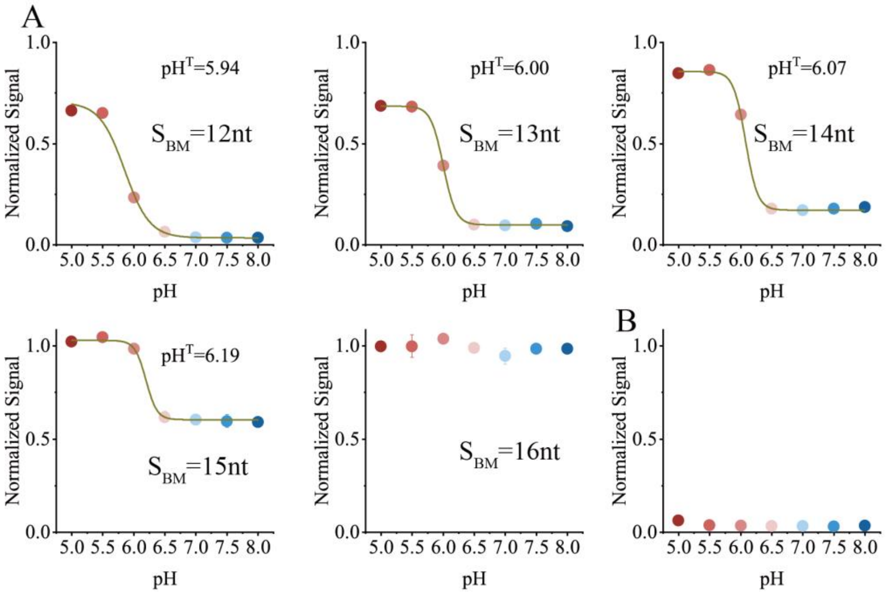

Figure S39. (A) Introduction of three C-A pairs with 8 nt toehold. (B) Fluorescence signals at different pH values without shadow strands.

	pH^T	1C-A 3'	1C-A 5'	2C-A 3'	2C-A 5'	2C-A 3'+5'	3C-A
S/nt	13	5.74	5.84	5.78	5.90	5.86	6.00
S/nt	14	5.81	5.88	5.86	6.00	5.94	6.07
S/nt	15	5.92	6.03	6.05	6.21	6.21	6.19

Figure S40. The pH^T changes with different A^+-C pairs.

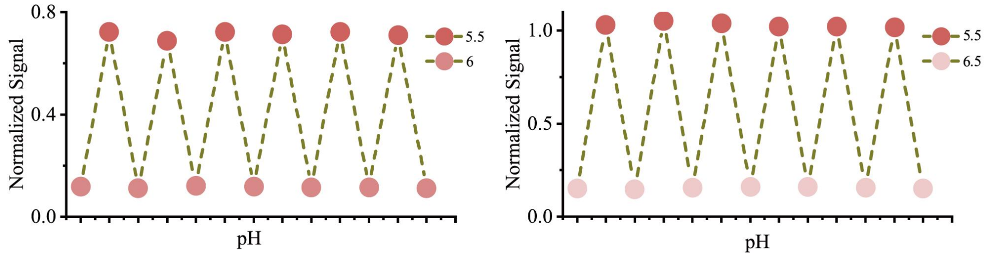

Figure S41. Resetability experiments with C-A pairs at different pH values. Left figure is resetability of 20-nt S with two A^+-C pairs and 8 nt toehold. Reft figure is resetability of 22-nt S with two A^+-C pairs and 8 nt toehold.

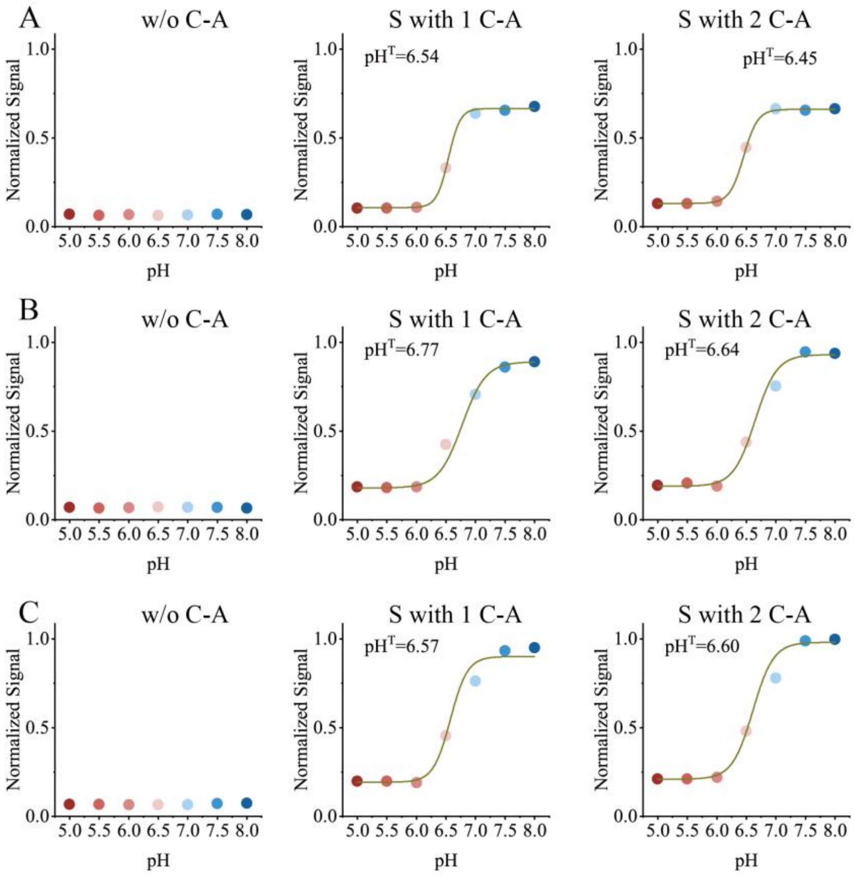

Figure S42. In HS system, signals at different pH values with different forward-to-reverse toehold differences: (A) 0 nt, (B) 1 nt, and (C) 2 nt.

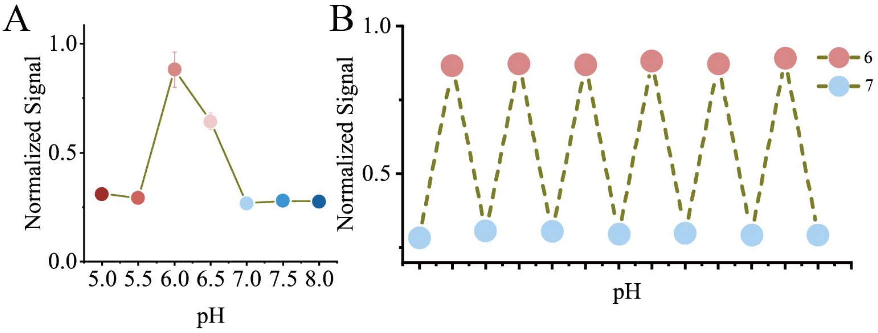

Figure S43. (A) Signals for HS with 16 nt branch migration and 8nt toehold at different pH values. (B) Resetability of the mildly acidic pH-sensitive design between pH 6.0 and pH 7.0.

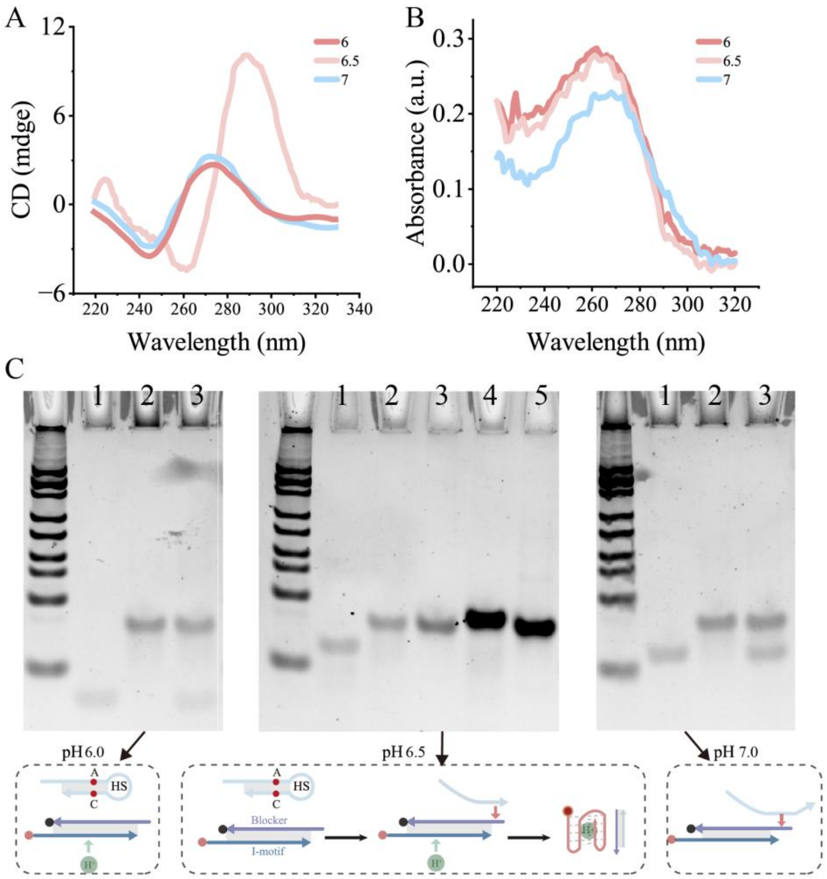

Figure S44. (A)CD of SHADE.The i-motif showed a positive peak at 288 nm at pH 6.5 and a negative peak at 263 nm, which is the characteristic spectrum of i-motif. (B)UV of SHADE. The absorbances at 265 nm and 295 nm are respectively characterized by hypochromism and hyperchromic effect as the pH dropped from 6 or 7 to 6.5. (C)PAGE characterization of SHADE. At pH 6.0, lane1: HS; lane2: duplex formed by i-motif-forming sequence and blocker; lane3: HS+duplex formed by i-motif-forming sequence and blocker. At pH 6.5, lane1: HS; lane2: duplex formed by i-motif-forming sequence and blocker; lane3: i-motif-forming sequence; lane4: duplex formed by HS and blocker; lane5: HS+duplex formed by i-motif-forming sequence and blocker. At pH 7.0, lane1: HS; lane2: duplex formed by i-motif-forming sequence and blocker; lane3: HS+duplex formed by i-motif-forming sequence and blocker. Only when the pH is 6.5 do the reactants disappeared and new bands appeared.

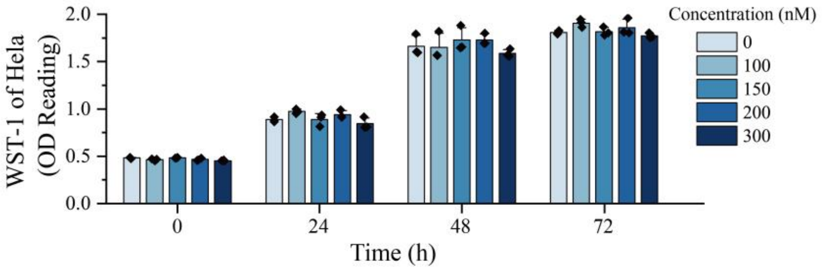

Figure S45. Cytotoxicity of SHADE. Absorbance of Hela cells treated with indicated concentrations of SHADE determined by WST-1.

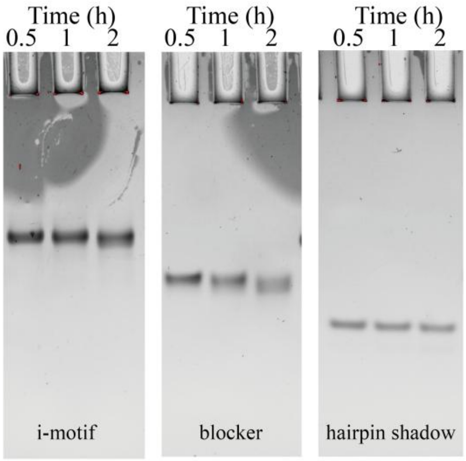

Figure S46. Serum stability test of SHADE. PAGE analysis of i-motif, blocker and shadow after incubation in 10% FBS for indicated time.

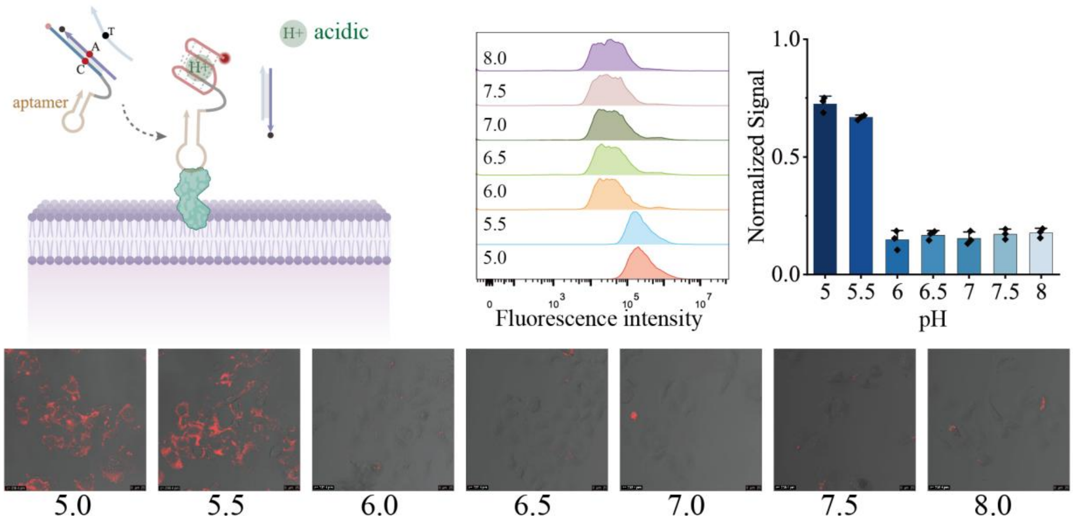

Figure S47. Cell imaging of shadow strands with 12nt branch migration and 8nt toehold.

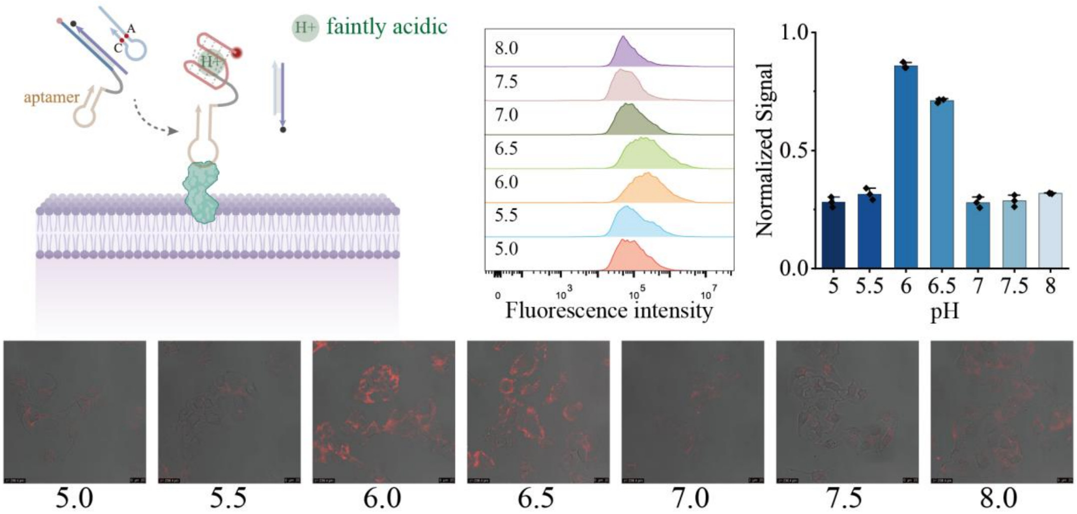

Figure S48. Cell imaging of hairpin shadow with 16nt branch migration and 8nt toehold.

Table S1. The sequences of DNA used in Figure2 and FigureS3-S31 and S33. (Ba-tb means that the hybridization length of i-motif and blocker is a nt and the forward toehold length is b nt. Sc-tb means that the branch migration domain is c nt and the forward toehold length is b nt. The forward toehold is marked in red.**)**

Name	Sequence (5'-3')
i-motif	/Cy5/ CCCCTAACCCCTAACCCCTAACCCC
B25-t0	GGGGTTAGGGGTTAGGGGTTAGGGG /BHQ2/
B24-t0	GGGGTTAGGGGTTAGGGGTTAGGG /BHQ2/
B23-t0	GGGGTTAGGGGTTAGGGGTTAGG /BHQ2/
B22-t0	GGGGTTAGGGGTTAGGGGTTAG /BHQ2/
B21-t0	GGGGTTAGGGGTTAGGGGTTA /BHQ2/
B20-t0	GGGGTTAGGGGTTAGGGGTT /BHQ2/
B19-t0	GGGGTTAGGGGTTAGGGGT /BHQ2/
B18-t0	GGGGTTAGGGGTTAGGGG /BHQ2/
B17-t0	GGGGTTAGGGGTTAGGG /BHQ2/
B16-t0	GGGGTTAGGGGTTAGG /BHQ2/
B15-t0	GGGGTTAGGGGTTAG /BHQ2/
S24-t0	CCCTAACCCCTAACCCCTAACCCC
S23-t0	CCTAACCCCTAACCCCTAACCCC
S22-t0	CTAACCCCTAACCCCTAACCCC
S21-t0	TAACCCCTAACCCCTAACCCC
S24-t0	AACCCCTAACCCCTAACCCC
S19-t0	ACCCCTAACCCCTAACCCC
S18-t0	CCCCTAACCCCTAACCCC
S17-t0	CCCTAACCCCTAACCCC
S16-t0	CCTAACCCCTAACCCC
S15-t0	CTAACCCCTAACCCC
S14-t0	TAACCCCTAACCCC
B25-t4	AGAAGGGGTTAGGGGTTAGGGGTTAGGGG /BHQ2/
B24-t4	AGAAGGGGTTAGGGGTTAGGGGTTAGGG /BHQ2/
B23-t4	AGAAGGGGTTAGGGGTTAGGGGTTAGG /BHQ2/
B22-t4	AGAAGGGGTTAGGGGTTAGGGGTTAG /BHQ2/
B21-t4	AGAAGGGGTTAGGGGTTAGGGGTTA /BHQ2/
B20-t4	AGAAGGGGTTAGGGGTTAGGGGTT /BHQ2/
B19-t4	AGAAGGGGTTAGGGGTTAGGGGT /BHQ2/
B18-t4	AGAAGGGGTTAGGGGTTAGGGG /BHQ2/

B17-t4	AGAAGGGGTTAGGGGTTAGGG /BHQ2/
B16-t4	AGAAGGGGTTAGGGGTTAGG /BHQ2/
B15-t4	AGAAGGGGTTAGGGGTTAG /BHQ2/
S18-t4	CCCCTAACCCCTAACCCCTTCT
S17-t4	CCCTAACCCCTAACCCCTTCT
S16-t4	CCTAACCCCTAACCCCTTCT
S15-t4	CTAACCCCTAACCCCTTCT
S14-t4	TAACCCCTAACCCCTTCT
S13-t4	AACCCCTAACCCCTTCT
S12-t4	ACCCCTAACCCCTTCT
S11-t4	CCCCTAACCCCTTCT
S10-t4	CCCTAACCCCTTCT
S9-t4	CCTAACCCCTTCT
B25-t6	GGAGAAGGGGTTAGGGGTTAGGGGTTAGGGG /BHQ2/
B24-t6	GGAGAAGGGGTTAGGGGTTAGGGGTTAGGG /BHQ2/
B23-t6	GGAGAAGGGGTTAGGGGTTAGGGGTTAGG /BHQ2/
B22-t6	GGAGAAGGGGTTAGGGGTTAGGGGTTAG /BHQ2/
B21-t6	GGAGAAGGGGTTAGGGGTTAGGGGTTA /BHQ2/
B20-t6	GGAGAAGGGGTTAGGGGTTAGGGGTT /BHQ2/
B19-t6	GGAGAAGGGGTTAGGGGTTAGGGGT /BHQ2/
B18-t6	GGAGAAGGGGTTAGGGGTTAGGGG /BHQ2/
B17-t6	GGAGAAGGGGTTAGGGGTTAGGG /BHQ2/
B16-t6	GGAGAAGGGGTTAGGGGTTAGG /BHQ2/
B15-t6	GGAGAAGGGGTTAGGGGTTAG /BHQ2/
S18-t6	CCCCTAACCCCTAACCCCTTCTCC
S17-t6	CCCTAACCCCTAACCCCTTCTCC
S16-t6	CCTAACCCCTAACCCCTTCTCC
S15-t6	CTAACCCCTAACCCCTTCTCC
S14-t6	TAACCCCTAACCCCTTCTCC
S13-t6	AACCCCTAACCCCTTCTCC
S12-t6	ACCCCTAACCCCTTCTCC
S11-t6	CCCCTAACCCCTTCTCC
S10-t6	CCCTAACCCCTTCTCC
S9-t6	CCTAACCCCTTCTCC
S8-t6	CTAACCCCTTCTCC

S7-t6	TAACCCCTTCTCC
S6-t6	AACCCCTTCTCC
B25-t8	GAGGAGAAGGGGTTAGGGGTTAGGGGTTAGGGG /BHQ2/
B24-t8	GAGGAGAAGGGGTTAGGGGTTAGGGGTTAGGG /BHQ2/
B23-t8	GAGGAGAAGGGGTTAGGGGTTAGGGGTTAGG /BHQ2/
B22-t8	GAGGAGAAGGGGTTAGGGGTTAGGGGTTAG /BHQ2/
B21-t8	GAGGAGAAGGGGTTAGGGGTTAGGGGTTA /BHQ2/
B20-t8	GAGGAGAAGGGGTTAGGGGTTAGGGGTT /BHQ2/
B19-t8	GAGGAGAAGGGGTTAGGGGTTAGGGGT /BHQ2/
B18-t8	GAGGAGAAGGGGTTAGGGGTTAGGGG /BHQ2/
B17-t8	GAGGAGAAGGGGTTAGGGGTTAGGG /BHQ2/
B16-t8	GAGGAGAAGGGGTTAGGGGTTAGG /BHQ2/
B15-t8	GAGGAGAAGGGGTTAGGGGTTAG /BHQ2/
S18-t8	CCCCTAACCCCTAACCCCTTCTCCTC
S17-t8	CCCTAACCCCTAACCCCTTCTCCTC
S16-t8	CCTAACCCCTAACCCCTTCTCCTC
S15-t8	CTAACCCCTAACCCCTTCTCCTC
S14-t8	TAACCCCTAACCCCTTCTCCTC
S13-t8	AACCCCTAACCCCTTCTCCTC
S12-t8	ACCCCTAACCCCTTCTCCTC
S11-t8	CCCCTAACCCCTTCTCCTC
S10-t8	CCCTAACCCCTTCTCCTC
S9-t8	CCTAACCCCTTCTCCTC
S8-t8	CTAACCCCTTCTCCTC
S7-t8	TAACCCCTTCTCCTC
S6-t8	AACCCCTTCTCCTC
S5-t8	ACCCCTTCTCCTC
S4-t8	CCCCTTCTCCTC
B25-t10	AGGAGGAGAAGGGGTTAGGGGTTAGGGGTTAGGGG /BHQ2/
B24-t10	AGGAGGAGAAGGGGTTAGGGGTTAGGGGTTAGGG /BHQ2/
B23-t10	AGGAGGAGAAGGGGTTAGGGGTTAGGGGTTAGG /BHQ2/
B22-t10	AGGAGGAGAAGGGGTTAGGGGTTAGGGGTTAG /BHQ2/
B21-t10	AGGAGGAGAAGGGGTTAGGGGTTAGGGGTTA /BHQ2/

B20-t10	AGGAGGAGAAGGGGTTAGGGGTTAGGGGTT /BHQ2/
B19-t10	AGGAGGAGAAGGGGTTAGGGGTTAGGGGT /BHQ2/
B18-t10	AGGAGGAGAAGGGGTTAGGGGTTAGGGG /BHQ2/
B17-t10	AGGAGGAGAAGGGGTTAGGGGTTAGGG /BHQ2/
B16-t10	AGGAGGAGAAGGGGTTAGGGGTTAGG /BHQ2/
B15-t10	AGGAGGAGAAGGGGTTAGGGGTTAG /BHQ2/
S18-t10	CCCCTAACCCCTAACCCCTTCTCCTCCT
S17-t10	CCCTAACCCCTAACCCCTTCTCCTCCT
S16-t10	CCTAACCCCTAACCCCTTCTCCTCCT
S15-t10	CTAACCCCTAACCCCTTCTCCTCCT
S14-t10	TAACCCCTAACCCCTTCTCCTCCT
S13-t10	AACCCCTAACCCCTTCTCCTCCT
S12-t10	ACCCCTAACCCCTTCTCCTCCT
S11-t10	CCCCTAACCCCTTCTCCTCCT
S10-t10	CCCTAACCCCTTCTCCTCCT
S9-t10	CCTAACCCCTTCTCCTCCT
S8-t10	CTAACCCCTTCTCCTCCT
S7-t10	TAACCCCTTCTCCTCCT
S6-t10	AACCCCTTCTCCTCCT
S5-t10	ACCCCTTCTCCTCCT
S4-t10	CCCCTTCTCCTCCT
S3-t10	CCCTTCTCCTCCT
B25-t12	GAAGGAGGAGAAGGGGTTAGGGGTTAGGGGTTAGGGG /BHQ2/
B24-t12	GAAGGAGGAGAAGGGGTTAGGGGTTAGGGGTTAGGG /BHQ2/
B23-t12	GAAGGAGGAGAAGGGGTTAGGGGTTAGGGGTTAGG /BHQ2/
B22-t12	GAAGGAGGAGAAGGGGTTAGGGGTTAGGGGTTAG /BHQ2/
B21-t12	GAAGGAGGAGAAGGGGTTAGGGGTTAGGGGTTA /BHQ2/
B20-t12	GAAGGAGGAGAAGGGGTTAGGGGTTAGGGGTT /BHQ2/
B19-t12	GAAGGAGGAGAAGGGGTTAGGGGTTAGGGGT /BHQ2/
B18-t12	GAAGGAGGAGAAGGGGTTAGGGGTTAGGGG /BHQ2/
B17-t12	GAAGGAGGAGAAGGGGTTAGGGGTTAGGG /BHQ2/
B16-t12	GAAGGAGGAGAAGGGGTTAGGGGTTAGG /BHQ2/

B15-t12	GAAGGAGGAGAAGGGGTTAGGGGTTAG /BHQ2/
S16-t12	CCTAACCCCTAACCCCTTCTCCTCCTTC
S15-t12	CTAACCCCTAACCCCTTCTCCTCCTTC
S14-t12	TAACCCCTAACCCCTTCTCCTCCTTC
S13-t12	AACCCCTAACCCCTTCTCCTCCTTC
S12-t12	ACCCCTAACCCCTTCTCCTCCTTC
S11-t12	CCCCTAACCCCTTCTCCTCCTTC
S10-t12	CCCTAACCCCTTCTCCTCCTTC
S9-t12	CCTAACCCCTTCTCCTCCTTC
S8-t12	CTAACCCCTTCTCCTCCTTC
S7-t12	TAACCCCTTCTCCTCCTTC
S6-t12	AACCCCTTCTCCTCCTTC
S5-t12	ACCCCTTCTCCTCCTTC
S4-t12	CCCCTTCTCCTCCTTC
S3-t12	CCCTTCTCCTCCTTC

Table S2. The sequences of DNA used in FigureS2 and FigureS32. **(**The forward toehold is marked in red**)**

Nsubstrate	/Cy5/ ATGACCCTACCTACCTACCCTGCG
Nblocker	CACATCATCGCAGGGTAGGTAGGTAG /BHQ2/
Ninvader	CTACCCTGCGATGATGTG
C-S	/Cy5/ CCTCTAACTCTTAACTCTTAACTCC
C-B	GAGGAGAAGGAGTTAAGAGTTAAGAGTTAGA /BHQ2/
C-S/+3	CTCTTAACTCTTAACTCCTTCTCCTC
C-S/+2	TCTTAACTCTTAACTCCTTCTCCTC
C-S/+1	CTTAACTCTTAACTCCTTCTCCTC
C-S/+0	TTAACTCTTAACTCCTTCTCCTC
C-S/-1	TAACTCTTAACTCCTTCTCCTC
C-S/-2	AACTCTTAACTCCTTCTCCTC
C-S/-3	ACTCTTAACTCCTTCTCCTC

Table S3. The sequences of DNA used in Figure3A-3G and FigureS34-S41. (Ba-tb means that the hybridization length of i-motif and blocker is a nt and the forward toehold length is b nt. Sc-tb means that the branch migration domain is c nt and the forward toehold length is b nt. dM3 means there are d mismatched bases in 3', dM5 means there are d mismatched bases in 5', and dM35 means there are d mismatched bases each in 3'and 5'. The forward toehold is marked in red and the mutant bases are marked in green.**)**

B23-t9-1M3	GGAGGAGAAGGGGTTAGGGATTAGGGGTTAGG /BHQ2/
B23-t8-1M3	GAGGAGAAGGGGTTAGGGATTAGGGGTTAGG /BHQ2/
B23-t7-1M3	AGGAGAAGGGGTTAGGGATTAGGGGTTAGG /BHQ2/
S16-t8-1M3	CCTAATCCCTAACCCCTTCTCCTC
S15-t8-1M3	CTAATCCCTAACCCCTTCTCCTC
S14-t8-1M3	TAATCCCTAACCCCTTCTCCTC
S13-t8-1M3	AATCCCTAACCCCTTCTCCTC
S16-t7-1M3	CCTAATCCCTAACCCCTTCTCCT
S15-t7-1M3	CTAATCCCTAACCCCTTCTCCT
S14-t7-1M3	TAATCCCTAACCCCTTCTCCT
S13-t7-1M3	AATCCCTAACCCCTTCTCCT
S16-t9-1M3	CCTAATCCCTAACCCCTTCTCCTCC
S15-t9-1M3	CTAATCCCTAACCCCTTCTCCTCC
S14-t9-1M3	TAATCCCTAACCCCTTCTCCTCC
S13-t9-1M3	AATCCCTAACCCCTTCTCCTCC
B23-t9-1M5	GGAGGAGAAGGAGTTAGGGGTTAGGGGTTAGG /BHQ2/
B23-t8-1M5	GAGGAGAAGGAGTTAGGGGTTAGGGGTTAGG /BHQ2/
B23-t7-1M5	AGGAGAAGGAGTTAGGGGTTAGGGGTTAGG /BHQ2/
S17-t8-1M5	CCCTAACCCCTAACTCCTTCTCCTC
S16-t8-1M5	CCTAACCCCTAACTCCTTCTCCTC
S15-t8-1M5	CTAACCCCTAACTCCTTCTCCTC
S14-t8-1M5	TAACCCCTAACTCCTTCTCCTC
S13-t8-1M5	AACCCCTAACTCCTTCTCCTC
S17-t7-1M5	CCCTAACCCCTAACTCCTTCTCCT
S16-t7-1M5	CCTAACCCCTAACTCCTTCTCCT
S15-t7-1M5	CTAACCCCTAACTCCTTCTCCT
S14-t7-1M5	TAACCCCTAACTCCTTCTCCT
S13-t7-1M5	AACCCCTAACTCCTTCTCCT
S17-t9-1M5	CCCTAACCCCTAACTCCTTCTCCTCC
S16-t9-1M5	CCTAACCCCTAACTCCTTCTCCTCC
S15-t9-1M5	CTAACCCCTAACTCCTTCTCCTCC
S14-t9-1M5	TAACCCCTAACTCCTTCTCCTCC
S13-t9-1M5	AACCCCTAACTCCTTCTCCTCC
B23-t9-1M35	GGAGGAGAAGGAGTTAGGGATTAGGGGTTAGG

	/BHQ2/
B23-t8-1M35	GAGGAGAAGGAGTTAGGGATTAGGGGTTAGG /BHQ2/
B23-t7-1M35	AGGAGAAGGAGTTAGGGATTAGGGGTTAGG /BHQ2/
S16-t8-1M35	CCTAATCCCTAACTCCTTCTCCTC
S15-t8-1M35	CTAATCCCTAACTCCTTCTCCTC
S14-t8-1M35	TAATCCCTAACTCCTTCTCCTC
S13-t8-1M35	AATCCCTAACTCCTTCTCCTC
S16-t7-1M35	CCTAATCCCTAACTCCTTCTCCT
S15-t7-1M35	CTAATCCCTAACTCCTTCTCCT
S14-t7-1M35	TAATCCCTAACTCCTTCTCCT
S13-t7-1M35	AATCCCTAACTCCTTCTCCT
S16-t9-1M35	CCTAATCCCTAACTCCTTCTCCTCC
S15-t9-1M35	CTAATCCCTAACTCCTTCTCCTCC
S14-t9-1M35	TAATCCCTAACTCCTTCTCCTCC
S13-t9-1M35	AATCCCTAACTCCTTCTCCTCC
B23-t8-2M3	GAGGAGAAGGGGTTAGGAATTAGGGGTTAGG /BHQ2/
S16-t8-2M3	CCTAATTCCTAACCCCTTCTCCTC
S15-t8-2M3	CTAATTCCTAACCCCTTCTCCTC
S14-t8-2M3	TAATTCCTAACCCCTTCTCCTC
S13-t8-2M3	AATTCCTAACCCCTTCTCCTC
S12-t8-2M3	ATTCCTAACCCCTTCTCCTC
B23-t8-2M5	GAGGAGAAGGAATTAGGGGTTAGGGGTTAGG /BHQ2/
S16-t8-2M5	CCTAACCCCTAATTCCTTCTCCTC
S15-t8-2M5	CTAACCCCTAATTCCTTCTCCTC
S14-t8-2M5	TAACCCCTAATTCCTTCTCCTC
S13-t8-2M5	AACCCCTAATTCCTTCTCCTC
S12-t8-2M5	ACCCCTAATTCCTTCTCCTC
B23-t8-3M	GAGGAGAAGGGGTTAAAAGTTAGGGGTTAGG /BHQ2/
S16-t8-3M	CCTAACTTTTAACCCCTTCTCCTC
S15-t8-3M	CTAACTTTTAACCCCTTCTCCTC
S14-t8-3M	TAACTTTTAACCCCTTCTCCTC
S13-t8-3M	AACTTTTAACCCCTTCTCCTC
S12-t8-3M	ACTTTTAACCCCTTCTCCTC

Table S4. The sequences of DNA used in Figure3H-3N and FigureS42-44. ((HS-a(C-A)-b means there are a C-A mismatches in the hairpin shadows, and Δf_T-r_T is bnt. The forward toehold is marked in red and the C-A mismatch are marked in green. The stem of HS is underlined. The aptamer is marked in purple. "Alk" means alkaline response.)

Alk-S	/Cy5/ GCTCTACTCGCACTTTCTC
Alk-B	CGGTAGAAGAGAAAGTGCGAGTAGAGC /BHQ2/
HS-0(C-A)-0	CGCACTTTCTCTTCTACCGTATTTTCGGTAGAA
HS-1(C-A)-0	CGCACTTTCTCTTCTACCGTATTTTCGATAGAA
HS-2(C-A)-0	CGCACTTTCTCTTCTACCGTATTTTCGATAAAA
HS-0(C-A)-1	TCGCACTTTCTCTTCTACCGTATTTTCGGTAGAA
HS-1(C-A)-1	TCGCACTTTCTCTTCTACCGTATTTTCGATAGAA
HS-2(C-A)-1	TCGCACTTTCTCTTCTACCGTATTTTCGATAAAA
HS-0(C-A)-2	CTCGCACTTTCTCTTCTACCGTATTTTCGGTAGAA
HS-1(C-A)-2	CTCGCACTTTCTCTTCTACCGTATTTTCGATAGAA
HS-2(C-A)-2	CTCGCACTTTCTCTTCTACCGTATTTTCGATAAAA
Narrow-B	GAGGAGAAGGGGTTAGGGGTTAGGGGTTAGG /BHQ2/
Imotif-HS15	CTAACCCCTAACCCCTTCTCCTCTATTTTGAAAAGAA
Imotif-HS16	CCTAACCCCTAACCCCTTCTCCTCTATTTTGAAAAGAA

Table S5. Figure4-5 and FigureS45-48. (The mutant bases are marked in yellow. C-A mismatches are marked in green. "Alk" means alkaline response.)

Apt-imotif	/Cy5/CCCCTAACCCCTAACCCCTAACCCCAAA**ATCAGGCTGGATGGTAGCTCGGTCGGGGTGGGTGGGTTGGCAAGTCTGAT**
B	GAGGAGAAGGGGTTAGGGGTTAGGGGTTAGG /BHQ2/
S16-t8	CCTAACCCCTAACCCCTTCTCCTC
B23-t8-2M5	GAGGAGAAGGAATTAGGGGTTAGGGGTTAGG /BHQ2/
S16-t8-2M5	CCTAACCCCTAATTCCTTCTCCTC
Apt-Alk-S	/Cy5/GCTCTACTCGCACTTTCTCAAA**ATCAGGCTGGATGGTAGCTCGGTCGGGGTGGGTGGGTTGGCAAGTCTGAT**
Alk-B	CGGTAGAAGAGAAAGTGCGAGTAGAGC /BHQ2/
HS-2(C-A)-1	TCGCACTTTCTCTTCTACCGTATTTTCGATAAAA
Imotif-HS15	CTAACCCCTAACCCCTTCTCCTCTATTTTGAAAAGAA
Apt-Mimotif	CCC**GCTCTACTCGCACTTTCTC**CCCAAA**ATCAGGCTGGATGGTAGCTCGGTCGGGGTGGGTGGGTTGGCAAGTCTGAT**
$B_{Apt\text{-}imotif\text{-}M}$	GAGGAGAAGGGGAGAAAGTGCGAGTAGAGCG/BHQ2/

MApt-imotif	/Cy5/CCCCTAACCCCTAACCCCTAACCCCAAAATCATGCTGGATGTTAGCTCTGTCGGGGTGTGTGGGTTGTCAATTCTGAT

Table S6. Performance comparison

strategy	tunability	selectivity
SHADE	Yes	Yes (pH 6.5)
MAT-amp(1)	No	No
pH-Responsive DNA Motifs with General Sequence Compatibility(2)	No	No
64Cu-UPS(3)	No	No
detachable DNA circuit (4)	No	No
PINS(5)	No	No
pHSCsgc8 (6)	No	No
PEGMnCaP(7)	No	No
DCPA-H2O(8)	No	No
pH-Responsive Iron Oxide Nanocluster Assemblies(9)	No	No
Si-4Py(10)	Yes	No
Triplex pH nanoswitches(11)	Yes	No
UPS(12-15)	Yes	No
reversible self-assembly system(16)	Yes	No
Highly Responsive pH Sensors(17)	Yes	No
Entropy-based pH-dependent nanoswitch(18)	Yes	No

References

1. Di, Z., Lu, X., Zhao, J. *et al.* (2022) Mild Acidosis-Directed Signal Amplification in Tumor Microenvironment via Spatioselective Recruitment of DNA Amplifiers. *Angew. Chem., Int. Ed. Engl.*, **61**, e202205436. http://doi.org/10.1002/anie.202205436.
2. Fu, W., Tang, L., Wei, G. *et al.* (2019) Rational Design of pH-Responsive DNA Motifs with General Sequence Compatibility. *Angew. Chem., Int. Ed. Engl.*, **58**, 16405-16410. http://doi.org/10.1002/anie.201906972.
3. Huang, G., Zhao, T., Wang, C. *et al.* (2020) PET imaging of occult tumours by temporal integration of tumour-acidosis signals from pH-sensitive (64)Cu-labelled polymers. *Nat. Biomed. Eng.*, **4**, 314-324. http://doi.org/10.1038/s41551-019-0416-1.
4. Guo, Y., Yao, D., Zheng, B. *et al.* (2020) pH-Controlled Detachable DNA Circuitry and Its Application in Resettable Self-Assembly of Spherical Nucleic Acids. *ACS Nano*, **14**, 8317-8327. http://doi.org/10.1021/acsnano.0c02329.
5. Zhao, T., Huang, G., Li, Y. *et al.* (2016) A Transistor-like pH Nanoprobe for Tumour Detection and Image-guided Surgery. *Nat. Biomed. Eng.*, **1**. http://doi.org/10.1038/s41551-016-0006.
6. Zhang, J., Wang, D., Chen, H. *et al.* (2022) A pH-Responsive Covalent Nanoscale Device Enhancing Temporal and Force Stability for Specific Tumor Imaging. *Nano Lett.*, **22**, 9441-9449. http://doi.org/10.1021/acs.nanolett.2c03487.
7. Mi, P., Kokuryo, D., Cabral, H. *et al.* (2016) A pH-activatable nanoparticle with signal-amplification capabilities for non-invasive imaging of tumour malignancy. *Nat. Nanotechnol.*, **11**, 724-730. http://doi.org/10.1038/nnano.2016.72.
8. Wang, D.Y., Yang, G., van der Mei, H.C. *et al.* (2021) Liposomes with Water as a pH-Responsive Functionality for Targeting of Acidic Tumor and Infection Sites. *Angew. Chem., Int. Ed. Engl.*, **60**, 17714-17719. http://doi.org/10.1002/anie.202106329.
9. Lu, J., Sun, J., Li, F. *et al.* (2018) Highly Sensitive Diagnosis of Small Hepatocellular Carcinoma Using pH-Responsive Iron Oxide Nanocluster Assemblies. *J. Am. Chem. Soc.*, **140**, 10071-10074. http://doi.org/10.1021/jacs.8b04169.
10. Zhang, L.N., Chen, S.Y., Shi, L. *et al.* (2024) De Novo Construction of pKa - Tunable Xanthene Molecules for pH Sensitive Fluorescence Navigation. *Adv. Funct. Mater.*, **35**. http://doi.org/10.1002/adfm.202412595.
11. Idili, A., Vallée-Bélisle, A. and Ricci, F. (2014) Programmable pH-triggered DNA nanoswitches. *J. Am. Chem. Soc.*, **136**, 5836-5839. http://doi.org/10.1021/ja500619w.
12. Wang, Y., Zhou, K., Huang, G. *et al.* (2014) A nanoparticle-based strategy for the imaging of a broad range of tumours by nonlinear amplification of microenvironment signals. *Nat. Mater.*, **13**, 204-212. http://doi.org/10.1038/nmat3819.
13. Ma, X., Wang, Y., Zhao, T. *et al.* (2014) Ultra-pH-sensitive nanoprobe library with broad pH tunability and fluorescence emissions. *J. Am. Chem. Soc.*, **136**, 11085-11092. http://doi.org/10.1021/ja5053158.
14. Li, Y., Zhao, T., Wang, C. *et al.* (2016) Molecular basis of cooperativity in pH-triggered supramolecular self-assembly. *Nat. Commun.*, **7**, 13214. http://doi.org/10.1038/ncomms13214.

15. Pan, M., Zhao, R., Fu, C. *et al.* (2024) Tuning nanoparticle core composition drives orthogonal fluorescence amplification for enhanced tumour imaging. *Nat. Commun.*, **15**, 7824. http://doi.org/10.1038/s41467-024-52029-7.
16. Dong, B., Du, S., Wang, C. *et al.* (2019) Reversible Self-Assembly of Nanoprobes in Live Cells for Dynamic Intracellular pH Imaging. *ACS Nano*, **13**, 1421-1432. http://doi.org/10.1021/acsnano.8b07054.
17. Nesterova, I.V. and Nesterov, E.E. (2014) Rational design of highly responsive pH sensors based on DNA i-motif. *J. Am. Chem. Soc.*, **136**, 8843-8846. http://doi.org/10.1021/ja501859w.
18. Mariottini, D., Idili, A., Nijenhuis, M.A.D. *et al.* (2019) Entropy-Based Rational Modulation of the pK(a) of a Synthetic pH-Dependent Nanoswitch. *J. Am. Chem. Soc.*, **141**, 11367-11371. http://doi.org/10.1021/jacs.9b04168.
